# Recent progress in experimental studies on the catalytic mechanism of cytochrome *c* oxidase

**DOI:** 10.3389/fchem.2023.1108190

**Published:** 2023-05-04

**Authors:** Atsuhiro Shimada, Tomitake Tsukihara, Shinya Yoshikawa

**Affiliations:** ^1^ Department of Applied Life Science, Faculty of Applied Biological Sciences, Gifu University, Gifu, Japan; ^2^ Department of Life Science, Graduate School of Science, University of Hyogo, Hyogo, Japan; ^3^ Institute for Protein Research, Osaka University, Osaka, Japan

**Keywords:** cytochrome *c* oxidase, X-ray crystal structure, proton-pump mechanism, O_2_ reduction mechanism, bioenergetics, enzyme reaction mechanism

## Abstract

Cytochrome *c* oxidase (CcO) reduces molecular oxygen (O_2_) to water, coupled with a proton pump from the N-side to the P-side, by receiving four electrons sequentially from the P-side to the O_2_-reduction site—including Fe_*a*3_ and Cu_B_—via the two low potential metal sites; Cu_A_ and Fe_*a*_. The catalytic cycle includes six intermediates as follows, R (Fe_*a*3_^2+^, Cu_B_^1+^, Tyr244OH), A (Fe_*a*3_^2+^-O_2_, Cu_B_^1+^, Tyr244OH), P_m_ (Fe_*a*3_^4+^ = O^2−^, Cu_B_^2+^-OH^−^, Tyr244O•), F (Fe_*a*3_^4+^ = O^2−^, Cu_B_^2+^-OH^-^, Tyr244OH), O (Fe_*a*3_^3+^-OH^-^, Cu_B_^2+^-OH^−^, Tyr244OH), and E (Fe_*a*3_^3+^-OH^-^, Cu_B_^1+^-H_2_O, Tyr244OH). CcO has three proton conducting pathways, D, K, and H. The D and K pathways connect the N-side surface with the O_2_-reduction site, while the H-pathway is located across the protein from the N-side to the P-side. The proton pump is driven by electrostatic interactions between the protons to be pumped and the net positive charges created during the O_2_ reduction. Two different proton pump proposals, each including either the D-pathway or H-pathway as the proton pumping site, were proposed approximately 30 years ago and continue to be under serious debate. In our view, the progress in understanding the reaction mechanism of CcO has been critically rate-limited by the resolution of its X-ray crystallographic structure. The improvement of the resolutions of the oxidized/reduced bovine CcO up to 1.5/1.6 Å resolution in 2016 provided a breakthrough in the understanding of the reaction mechanism of CcO. In this review, experimental studies on the reaction mechanism of CcO before the appearance of the 1.5/1.6 Å resolution X-ray structures are summarized as a background description. Following the summary, we will review the recent (since 2016) experimental findings which have significantly improved our understanding of the reaction mechanism of CcO including: 1) redox coupled structural changes of bovine CcO; 2) X-ray structures of all six intermediates; 3) spectroscopic findings on the intermediate species including the Tyr244 radical in the P_m_ form, a peroxide-bound form between the A and Pm forms, and F_r_, a one-electron reduced F-form; 4) time resolved X-ray structural changes during the photolysis of CO-bound fully reduced CcO using XFEL; 5) a simulation analysis for the Pm→Pr→F transition.

## 1 Introduction

Cytochrome *c* oxidase (CcO) is the terminal oxidase of cell respiration which reduces molecular oxygen (O_2_), coupled with proton-pumping from the N-side (inside) to the P-side (outside). CcO contains four redox active sites as its active center, designated as Cu_A_, Fe_*a*_, Cu_B_, and Fe_*a*3_, each of which reversibly accepts one electron (Mochizuki et al., 1999). The Cu_B_ and Fe_*a*3_ sites form an O_2_-reduction site (BNC) which is capable of accepting O_2_ to Fe_*a*3_ when both metal sites are in the reduced state (Dodson et al., 1996) and of reducing the O_2_ completely to 2H_2_O. The four electron equivalents for the O_2_-reduction are sequentially transferred from cytochrome *c* in the P-side to the BNC via the low potential metal sites, Cu_A_ and heme *a*, coupled with the transfer of one proton from the N-side to the O_2_-reduction site. This proton-coupled electron transfer creates a membrane potential equivalent to that created by active transport of one proton across the membrane. Furthermore, each of the four proton-coupled electron transfers is coupled with one proton pump from the N-side to the P-side giving a proton gradient across the membrane. The proton motive force (pmf) composed of the membrane potential and the proton gradient is utilized for ATP production by ATP synthase (Yoshikawa and Shimada, 2015; Wikström et al., 2018). In our view, the progress of the investigation of the CcO reaction mechanism has been critically rate-limited by the improvement of the resolution of the X-ray crystal structure. Thus, we will first summarize the progress in the experimental investigation of CcO, reaching a resolution of 1.8–2.0 Å, followed by reviewing the experimental findings since the appearance of the 1.5/1.6 Å resolution X-ray structures of the oxidized/reduced bovine CcO.

## 2 A summary of the experimental studies on the reaction mechanism of CcO, reaching an X-ray structural resolution of 1.8–2.0 Å

### 2.1 O_2_ reduction mechanism

**FIGURE 1:**
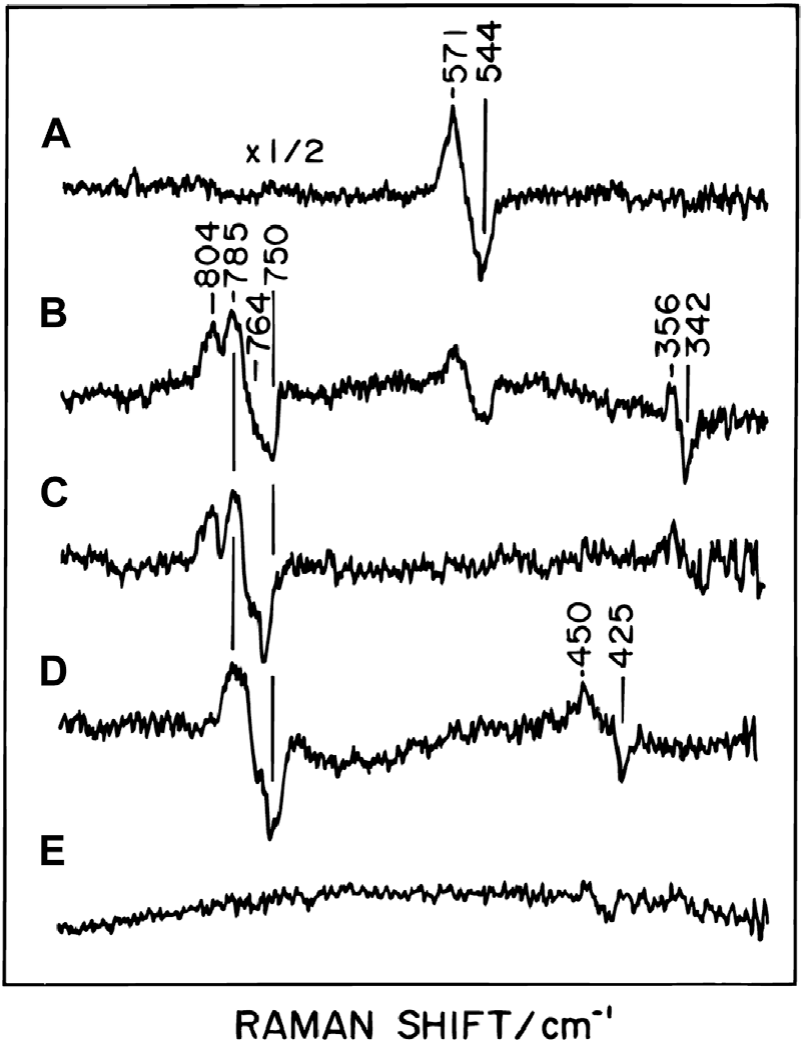
Time-resolved resonance Raman difference spectra of reaction intermediates of bovine heart CcO. The Raman difference spectra have been determined by subtracting the spectrum of the corresponding ^18^O_2_ derivative from the spectrum of the ^16^O_2_ derivative at each delay time, excited at a wavelength of 423 nm at 3°C. Positive and negative peaks denote the contributions of ^16^O_2_ and ^18^O_2_ derivatives, respectively. The delay time after the initiation of the reaction is 0.1 **(A)**, 0.27 **(B)**, 0.54 **(C)**, 2.7 **(D)** and 5.4 ms**(E)**. Reprinted with permission from (Yoshikawa and Shimada, 2015). Copyright 2015 American Chemical Society.

It is well known that one-electron reduction of triplet oxygen (O_2_) is energetically unfavorable but a simultaneous two-electron reduction is very favorable (Caughey et al., 1976). Though one electron reduction of O_2_ is possible in various metalloproteins (Kim, et al., 2020), this intrinsic property of O_2_ critically contributes to facilitating the reversible O_2_-binding to hemoglobins and myoglobins and thus to stabilizing the O_2_-bound form, since the second electron donation to the O_2_- bound form is effectively blocked by the protein moiety. On the other hand, as mentioned above, the BNC of CcO has Cu_B_^1+^ as the second electron donor to the O_2_ molecule bound at Fe_*a*3_^2+^. Thus, this structure strongly suggests that in the initial intermediate of the O_2_ reduction process, a peroxide is bound at Fe_*a*3_^3+^. However, unexpectedly, time-resolved resonance Raman analyses showed that the initial intermediate had a band at 571 cm^−1^ as shown in Figure 1 (Ogura et al., 1993; Ogura et al., 1996). This result indicates that the initial intermediate is an O_2_-bound form closely similar to those of oxygenated hemoglobins and myoglobins, that is, Fe_*a*3_^2+^-O_2_. This intermediate is designated as the A form. The resonance Raman band of the second intermediate was at 804 cm^−1^ (Figure 1). At this stage of the O_2_ reduction reaction, the bound O_2_ is completely reduced to 2 oxide ions (2O^2−^) giving Fe_*a*3_^4+^ = O^2−^ and Cu_B_^2+^-OH^−^. This form is designated as P_m_. For this process, two electrons are taken up from Fe_*a*3_^2+^, and one from Cu_B_^1+^. It has been proposed that the fourth electron and one proton are from Tyr244, giving the Tyr244 neutral radical, though without clear experimental confirmation (Yoshikawa and Shimada, 2015). In the normal catalytic turnover, after P_m_ formation, four electron equivalents are sequentially transferred from cytochrome *c* via the low potential sites, coupled with proton uptake from the N-side to the BNC, as described above, giving four intermediate forms designated as F, O, E, and R. The time-resolved resonance Raman analysis identified the ferryl oxide structure in the F-form giving the 785 cm^−1^ band (Figure 1), suggesting the elimination of the neutral radical in Tyr244O• to form Tyr244OH by proton-coupled electron transfer. The resonance Raman analyses showed that in the F to O transition, driven by the second proton-coupled electron transfer, the ferryl oxide structure (Fe_*a*3_^4+^ = O^2−^) was reduced to the Fe_*a*3_^3+^-OH^-^ structure, giving the 451 cm^−1^ band (Figure 1). Absorption spectral and electrometrical studies on the process O→E showed that Cu_B_^2+^-OH^−^ in the O state had extremely high electron affinity (Belevich, et al., 2007). It has been proposed that the E-form had Cu_B_^1+^-H_2_O and Fe_*a*3_^3+^-OH^-^ in the BNC. In the R-form, both metal sites in the BNC are in the ligand-free reduced state (Yoshikawa and Shimada, 2015). The absorption spectral peaks of the A, P_m_, and F forms are at 590, 607, and 580, respectively.

In the reaction between the fully reduced CcO and O_2_, the 607 nm species appears following the formation of the A-form, as quickly as oxidation of the reduced heme *a*. This 607 nm species gives the Raman band at 785 cm^−1^, not at 804 cm^−1^. Thus, the Raman band position suggests that this species is the one-electron reduced form of P_m_, while its absorption band is identical to that of P_m_. This species is designated as P_r_ (Morgan, et al., 2001; Han, et al., 2000). The oxidation and ligand-binding states of the BNC of the P_r_-form are identical to those of the F-form except for the protonation state of Tyr244 (Fe_*a*3_^4+^ = O^2−^, Cu_B_^2+^-OH^−^, Tyr244O^−^). This form is transformed to the F-form (Fe_*a*3_^4+^ = O^2−^, Cu_B_^2+^-OH^−^, Tyr244OH) by receiving a proton from the N-side. In this Pr→F transition, a single protonation induces a large absorption spectral change, from the 607 nm peak to the 580 nm peak (Jünemann, et al., 2000). The purified fully oxidized CcO has no proton pump function (Verkhovsky et al., 1999) in contrast to the fully oxidized form which appears under turnover conditions, that is, the O-form as described above. Thus, the CcO in the purified preparation is designated as the resting oxidized form.

### 2.2 Proton-conducting pathway and proton pump cycle

**FIGURE 2:**
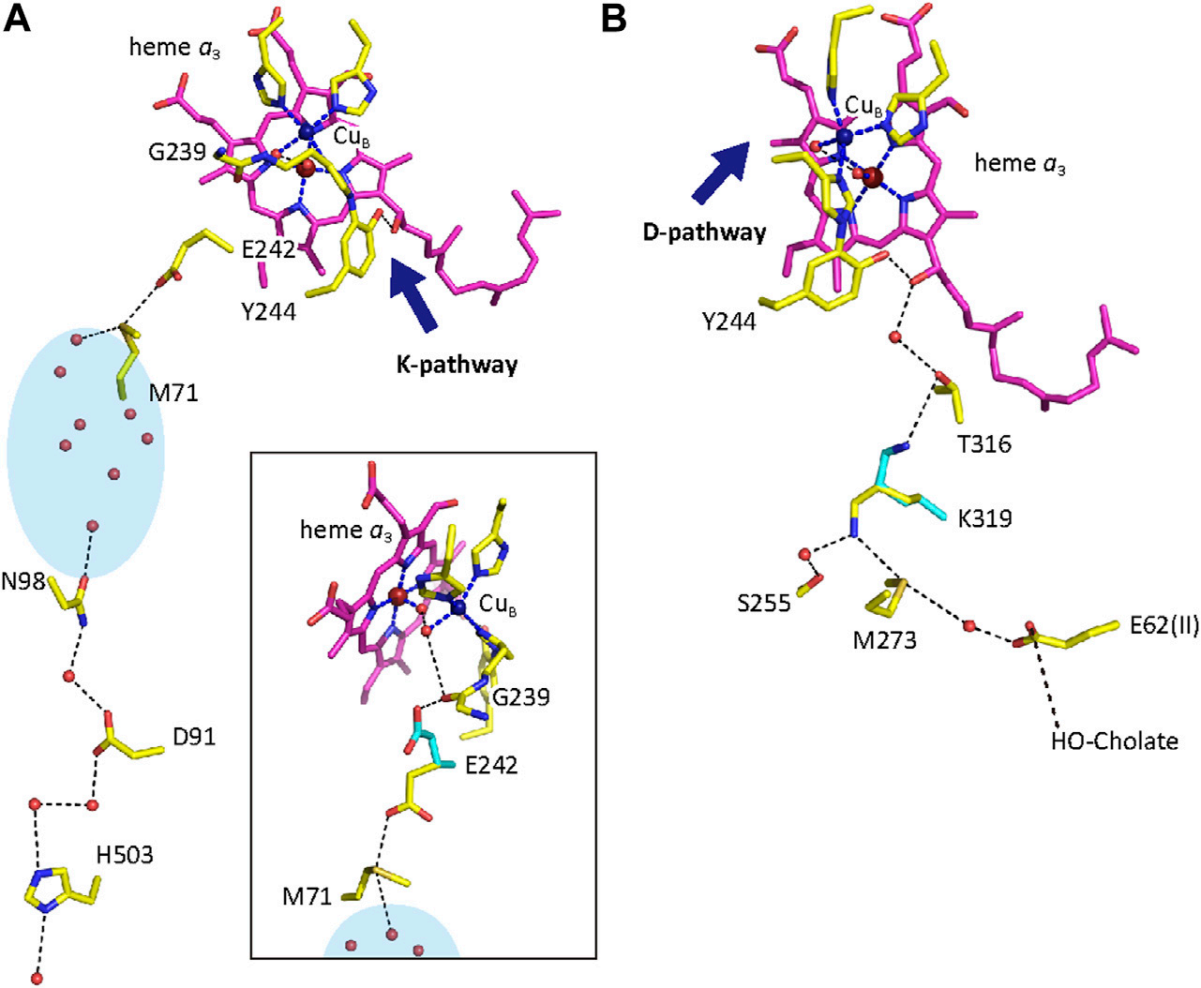
Atomic models of the D- and K-pathways. Purple models denote heme *a*_3_. The dark blue, red and beige portions of amino acids are nitrogen, oxygen and sulfur atoms, respectively. Dotted and broken lines are hydrogen and coordination bonds, respectively. The dark blue spheres and the large red spheres are the Cu_B_ and Fe_*a*3_ atoms, respectively. The two metal ions form the BNC. The D- and K-pathways are shown in panels **(A)** and **(B)**, respectively. The junction points for the K- and D-pathways are denoted by blue arrows in panels **(A)** and **(B)**, respectively. The inset in panel **(A)** shows a possible hydrogen-bond network from Glu242 to the BNC. The light-blue oval indicates a water cluster, including fixed water marked by red small spheres. The blue structures denote possible structural changes for transferring protons. His503, D91 and E62 are located at or near the N-side surface of CcO. The BNC receives protons for making waters from the N-side through the D and K pathways and electrons from heme *a* (not shown for the sake of simplicity). Reprinted with permission from (Yoshikawa and Shimada, 2015). Copyright 2015 American Chemical Society.

**FIGURE 3:**
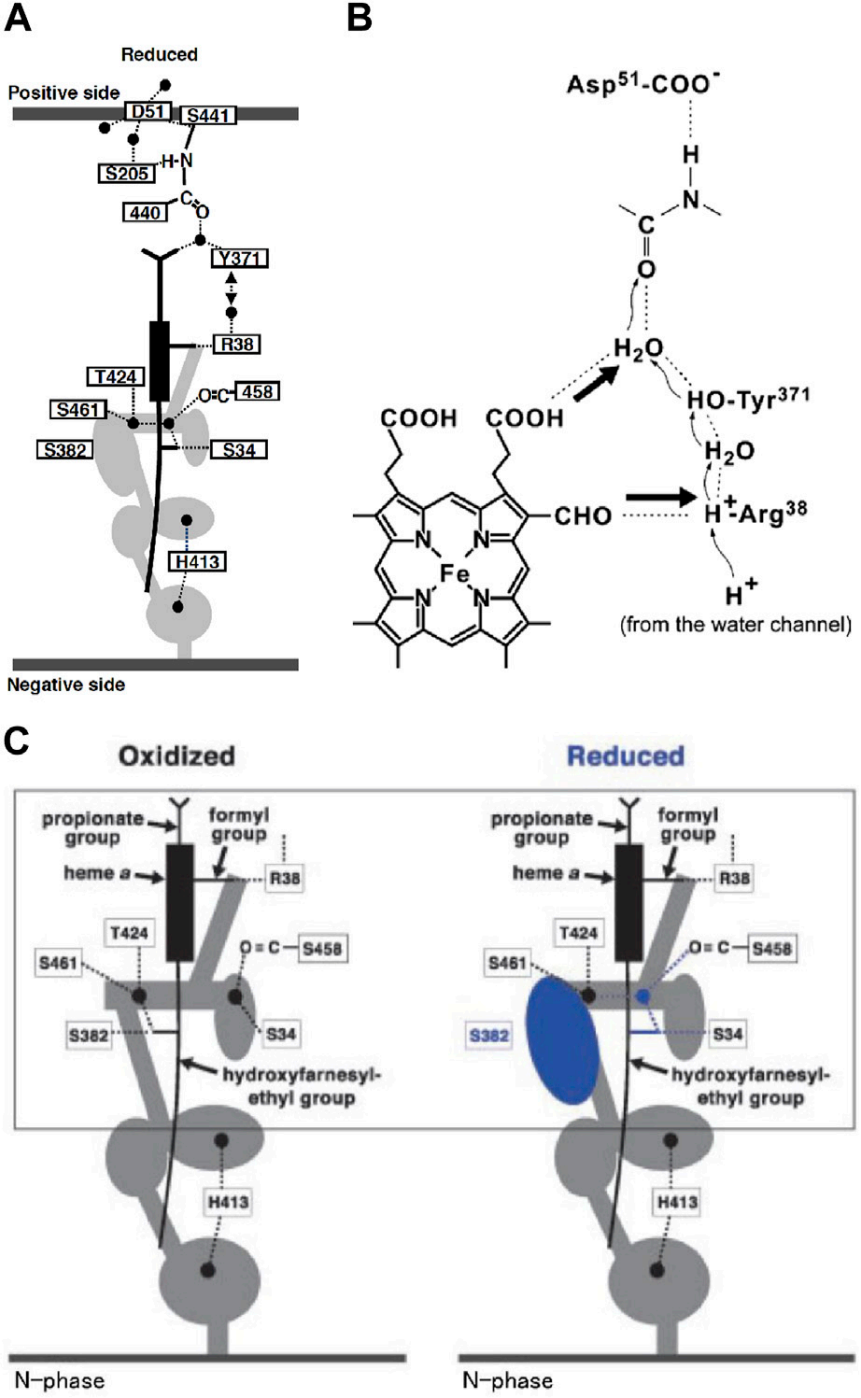
Schematic representation of the H-pathway of bovine heart CcO. **(A)** The filled circles denote fixed water molecules detectable in the X-ray structure. The water channel is represented by the gray area. The circle and ovals in the water channel denote the spaces which have capacity for trapping at least one water molecule. The side view of the heme *a* plane is shown as a rectangle, with sticks denoting the peripheral groups. The dotted lines represent hydrogen bonds. The two thick gray lines denote the mitochondrial inner membrane surfaces facing the P and N side phases, as labeled. **(B)** The interactions between the hydrogen-bond network in the H-pathway and heme *a*. The thick arrows denote possible electrostatic repulsion against proton transfer through the hydrogen-bond network. **(C)** The redox coupled open/closed transition in the water channel. The cavity colored in blue is eliminated upon oxidation. Reprinted with permission from (Yoshikawa and Shimada, 2015). Copyright 2015 American Chemical Society.

CcO has three proton conducting pathways, designated as D, K, and H. The D and K pathways connect the N-side with the O_2_ reduction site, as shown in Figure 2, indicating that protons for making water from the oxides, O^2−^, are transferred through the two pathways. The third proton-conducting pathway, the H-pathway, is extended from the N-side to the P-side including a water channel and a hydrogen bond network in tandem as shown in Figure 3A. The two heme *a*-peripheral groups are attached to the hydrogen bond network by forming two hydrogen bonds between the formyl group of heme *a* and Arg38 and between the D-ring propionate and a fixed water molecule in the channel (Figure 3B). The location of heme *a* suggests that proton transfer through the H-pathway is driven electrostatically by net positive charges created upon oxidation of heme *a* for the O_2_ reduction. It has been well-established that each of the four transitions, driven by the proton-coupled electron transfer from heme *a*, is coupled with pumping one proton equivalent across the mitochondrial (cytoplasmic) membrane (Bloch, et al., 2004). Furthermore, in each transition, one proton is released to the P-side and two protons are taken up from the N-side (Faxén, et al., 2005).

### 2.3 D-pathway mechanism

A D-pathway mutation for bacterial CcOs, Glu242Gln (in this article, numbering for bovine CcO is used unless otherwise noted.), blocks the P→F and F→O transitions, designated as the oxidative phase while a K-pathway mutation, Lys319Met, abolishes the two other transitions, O→E and E→R, designated as the reductive phase. Thus, it has been proposed that the D- and K-pathways transfer protons for the proton-coupled electron transfers in the oxidative and reductive phases, respectively (Yoshikawa and Shimada, 2015; Wikström et al., 2018). A mutation of the D-pathway, Asn98Asp, in bacterial CcO (Figure 2) abolishes its proton pump activity without affecting normal O_2_-reduction activity (Pawate, et al., 2002). One of the simplest interpretations for these results is that D-pathway transfers both protons for pumping as well as for making waters. This proposal is designated as the D-pathway mechanism in this review. As described in Figure 2, Glu242 is located on the P-side end of the D-pathway. The X-ray structure indicates a space connecting from Glu242 to the O_2_-reduction site and to a propionate group of the D-ring of heme *a*_3_. This space allows structural changes in the Glu242 side chain as schematically shown in Figure 2A inset. From this structure, the D-pathway mechanism proposes that Glu242 is the branch point for the transfers of protons for making water to the BNC, and of the pumping protons to the propionate group or a proton acceptable group near the propionate, the proton-loading site (PLS) (Belevich et al., 2006). Glu242 at first transfers pumping protons to the PLS and then transfers protons for making water to the BNC. The latter proton transfer ejects protons on the PLS to the P-side by electrostatic repulsion (Yoshikawa and Shimada, 2015; Wikström et al., 2018).

The PLS has not been identified experimentally, due to the strong mutual coupling between all potential PLSs. However, a simulation analysis suggested His291 and the two propionates of heme *a*_3_ as the main candidates (Popovic, 2013). The location of these putative PLS indicates no structural barrier against the O_2_-reduction site for avoiding direct proton transfer from PLS to the BNC which dissipates the proton pump energy. Thus, the proton transfer from Glu242 to the PLS must be much faster than that from Glu242 to the BNC, which is designated as the “kinetic gating”. It has been suggested that changes in hydration of the space connecting Glu242, the putative PLS and the BNC, in which no fixed water molecule is detectable in the X-ray structure, control the rate of the proton transfer inside the space for facilitating the kinetic gating (Popovic and Stuchebrukhov, 2012). Consistently, the side chain of Glu242 in a decoupled mutation (Asn98Asp) for the *Paracoccus denitrificans* CcO influenced the X-ray structure, giving two orientations for Glu242, one closely similar to that of the wild type CcO, namely, downward toward the entrance of the D-channel, and the other upward toward the BNC (Durr, et al., 2008). Possible exits for pumping protons from the PLS and product water molecules from the BNC have been proposed on the P-side surface of CcO by simulation analyses, suggesting multiple pathways (Popovic and Stuchebrukhov, 2005; Cai, et al., 2018). These exit pathways must block proton back leak from the P-side which is highly exergonic. Since no structural change has been reported, hydration state changes have been proposed in these possible pathways (Cai, et al., 2018).

**FIGURE 4:**
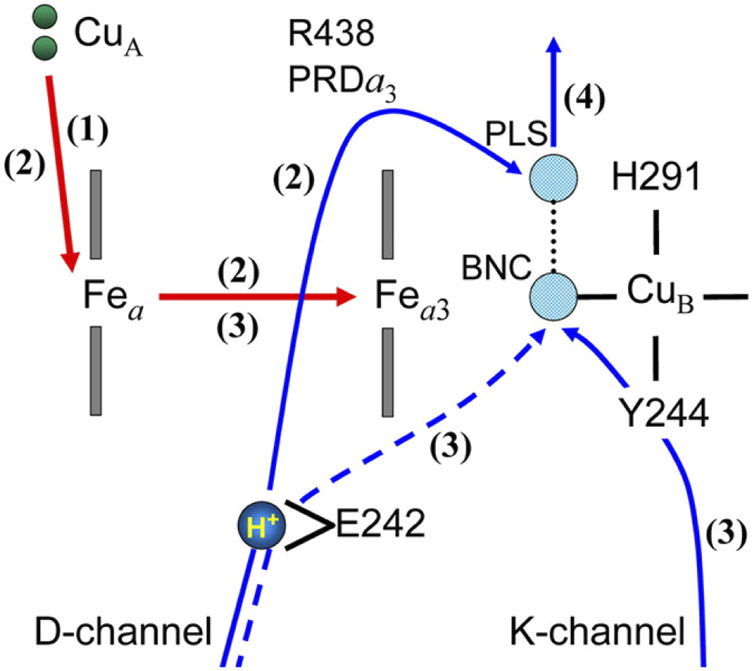
A schematic interpretation for the electrometric and spectroscopic results measured for the O→E transition (Belevich, et al., 2007). The numbers in parentheses denote the order of the kinetic phases observed. The red and blue arrows indicate the electron and proton transfers. The dotted blue arrow denotes the transfer of the water forming protons in the Pm→F and F→O transitions. The BNC denotes the OH^−^ ligand of Cu_B_ in this figure. The PLS is His291 liganded to Cu_B_ or adjacent propionic groups (PrA and PrD) of heme *a*_3_ in other theoretical models. E242 is Glu242, which has two possible conformers, up and down. Reprinted with permission from (Popovic and Stuchebrukhov, 2012).

Electrometric and spectrophotometric changes in the O→E transition of *P. denitrificans* CcO (Belevich, et al., 2007) were analysed by a combined DFT/electronic approach using the X-ray structure of bovine heart CcO and a D-pathway mechanism including His 291 as its proton-loading site, as schematically illustrated in Figure 4 (Popovic and Stuchebrukhov, 2012). Following single electron injection by a laser excitation to reduce Cu_A_ instantaneously, four electrometric phases were identified. In phase 1 (10 µs) 70% of Fe*_a_* is reduced leaving 30% electron in Cu_A_, without any proton translocation. In phase 2 (150 µs), a proton-transfer from Glu242 to His291 (PLS) in Figure 4 occurs coupled with an electron transfer from Fe*_a_* to Fe_*a*3_. This protonation of PLS significantly increases the E_m_ of both Fe sites to provide 60% of Fe_*a*3_^2+^ and 40% of Fe_*a*_^2+^. In phase 3 (800 µs), electron transfer from Fe_*a*_^2+^ to Fe_*a*3_^3+^ triggers the water forming (chemical) proton transfer to OH^−^ ligand at Cu_B_ designated as BNC in Figure 4. (The chemical proton is from the K-pathway via Tyr244.) The electron transfer to Fe_*a*3_^3+^ greatly increase the electron affinity of Cu_B_ so that Cu_B_ takes up all electrons in the system. The protonation of OH^−^ ligand at Cu_B_ (BNC in Figure 4) decreases the pKa of the PLS to destabilize the pump proton on the H291. In phase 4 (2.7 ms), the proton on the PLS is released to the P-side by the elctrostatic repulsion between the pumping proton and the chemical proton in the BNC and Tyr244 is reprotonated from the N-side as illustrated in Figure 4. The above interpretation for the experimental results is based on the calculated pK_a_s and E_m_s of the critical sites illustrated in Figure 4, based on the X-ray structure of CcO assuming the D-pathway mechanism as described above. Namely, the experimental results strongly support the D-pathway model including His291 as the proton-loading site. It is obvious that various electrostatic interactions between these protons and electrons are critical for the highly efficient energy coupling in CcO.

The following mutation results perhaps are the strongest experimental findings for the D-pathway mechanism in which both chemical and pump protons are transferred through the D-pathway: Asp91Asn mutation abolishes the turnover activity of CcO completely by inhibiting the F→O transition without affecting the Pr→F transition. As described in Figure 2, Asp91 is located near the N-side entrance of the D-pathway. Thus, in this mutant, Glu242 in the fully reduced state before O_2_ binding is protonated but Asn91 has no proton. The normal Pr→F transition detectable in this mutant suggests that the D-pathway above Asp91 has two protons, one for protonation of the O_2_ reduction site giving the Pr→F transition and the other for protonation of the PLS. However, in the Asp91Asn mutant, which blocks proton transfer from the N-side, the third proton for the F→O transition is not available, so that the F→O transition is completely inhibited. The existence of the second proton donor site above Asp91 has been proposed by a double mutation, Asp91Asn/Tyr19Phe. The well-conserved Tyr19 is located in the D-pathway between Asp91 and Glu242. The double mutant blocks the transitions, Pr→F (and thus F→O), whereas the vectorial proton transfer coupled with A→Pr transition is not blocked (Belevich, et al., 2010). This result strongly suggests that the two proton equivalents are loaded at Glu242 and Tyr19. However, it has been reported that the vectorial proton translocation coupled with A→Pr transition, which is blocked by the Glu242Gln mutation, is blocked also by a K-pathway mutation, Lys319Met (Lepp, et al., 2008). An alternative interpretation for this finding would be that Glu242 as well as Lys319 do not supply pumping protons to the PLS, and that the pumping-protons to the PLS are transferred through a proton-conducting pathway other than the D-pathway. However, the mutants, Glu242Gln and Lys319Met, block the proton translocation to the loading site by inactivating the proton-conducting pathway other than the D-pathway. Furthermore, Tyr19 does not transfer any protons to Glu242 but Phe19 abolishes the proton transfer function of Glu242 to drive the Pr→F transition. Thus, these mutation results are not conclusive experimental results for proving that D-pathway transports both pump and chemical protons.

### 2.4 H-pathway mechanism

As shown in Figure 3A, the hydrogen bond network of the H-pathway has Asp51 and Arg38 on the P-side and N-side ends, respectively, and a peptide bond between Tyr440 and Ser441. It has been shown experimentally that proton transfer through peptide bond is possible by forming an imidic acid intermediate (-C(OH) = N^+^H-) (Perrin, 1989). Heme *a* is attached to the hydrogen bond network by forming two hydrogen bonds as described in Figure 3B. The driving force for the proton transfer to the P-side is the electrostatic repulsion between the protons for pumping, transferred to the hydrogen bond network through the water channel, and the net positive charge of heme *a*, created upon its oxidation for reduction of O_2_ at the BNC. The water channel provides accessibility to the water molecules in the N-side phase to the Arg38 at the N-side end of the hydrogen bond network, and it has several cavities, each of which has enough space for keeping at least one water molecule. These cavities significantly increase the mobility of water molecules in the water channel since the mobility of these water molecules in the cavities is not limited by the thermal motion of the protein moiety surrounding the channel. The biggest cavity near the upper end of the water channel in the fully reduced state is eliminated by a structural change in Ser382 upon oxidation as shown in Figure 3C (Tsukihara, et al., 2003). This cavity elimination effectively blocks the water accessibility at least within the physiological timescale (∼ms) in the oxidized state, giving unidirectionality to the proton transfer driven by the electrostatic repulsion. The water channel structures in the oxidized and reduced states are designated as the “closed” and “open” states respectively (Figure 3C). The X-ray structures of various inhibitor-bound forms obtained before 2016 suggest that the water channel is in the open state only in the R-state which appears only once in the catalytic turnover. Thus, four equivalents of protons to be pumped should be stored above the water channel.

**FIGURE 5:**
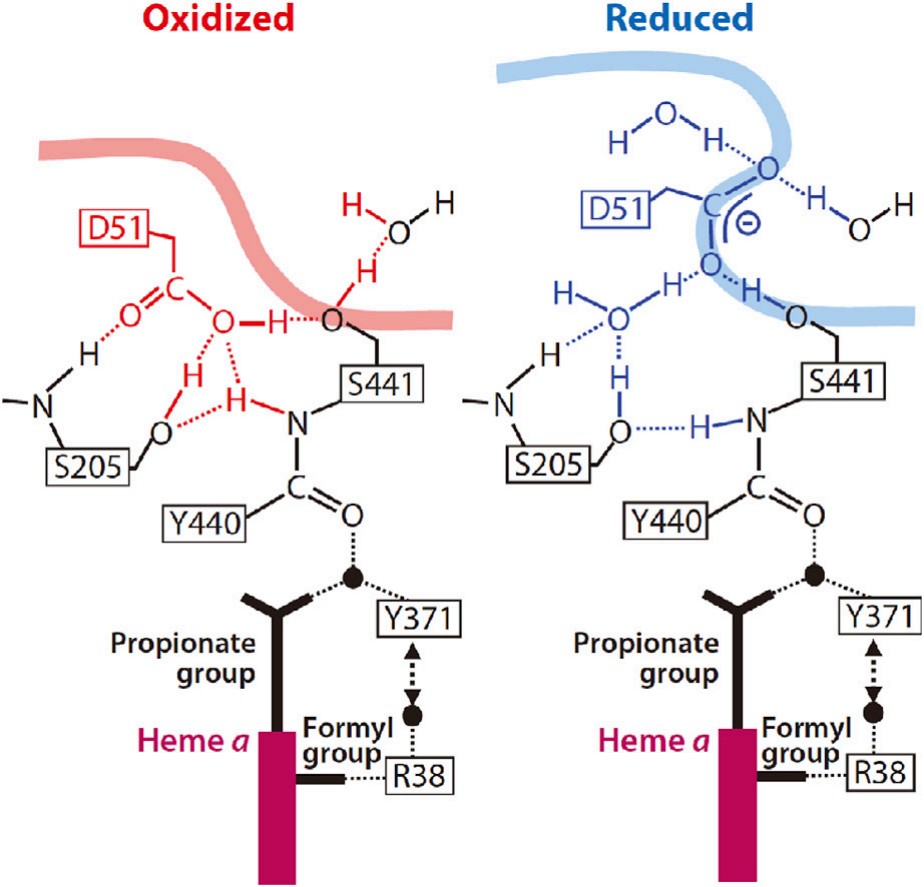
Schematic representation of the structural characteristics of Asp51 in the oxidized and reduced states. The smooth thick curves denote the molecular surface to which the water molecules in the P-phase have access. Conformational changes near Asp51 upon reduction of CcO are shown by the blue structure on the right. Reprinted with permission from (Yoshikawa and Shimada, 2015). Copyright 2015 American Chemical Society.

The X-ray structure of Asp51 schematically shown in Figure 5 indicates that the carboxyl group in the reduced state is exposed to the P-side phase, in other words, its effective dielectric environment is essentially identical to that in the aqueous solution, while in the fully oxidized state, the environment is essentially identical to that in a polar organic solvent (Tsukihara, et al., 2003). The pKa of acetic acid is increased to approximately 10 from 4.5 upon exchange of the solvent from water to methanol (Isaacs, 1995). The effective dielectric constant of the environment of the carboxyl group of Asp51 in the oxidized sate is definitely lower than that of methanol. Thus, the pKa of the carboxyl group of Asp51 must be significantly higher than 10. This structural change strongly suggests that Asp51 is protonated in the oxidized state and deprotonated in the reduced state. In contrast to the X-ray structural results, it has been shown that upon oxidation of heme *a*, protons are released from the bovine CcO to the P-side. (Capitanio et al., 2000a; Capitanio et al., 2000b; Forte et al., 2002). Thus, it was proposed 15 years ago that some proton-accepting groups located near Asp51 in the reduced state trap protons dissociated from Asp51 and release them to the P-side upon oxidation (Shimokata, et al., 2007). However, this proposal is still yet to be examined.

Mutation analyses on bovine CcO were performed by constructing an expression system for bovine heart subunit I in HeLa cells. The pumping proton transfer function of the hydrogen-bond network of the H-pathway was confirmed by Asp51Asn and Ser441Pro mutations both of which completely abolished the proton-pump function without influencing the O_2_ reduction function (Tsukihara et al., 2003; Shimokata, et al., 2007). A closely similar phenotype was obtained for a double mutation using bulkier residues, Val386Leu/Met390Trp, for the water channel elimination (Shimokata, et al., 2007). These results confirm strongly the proton pump function of the H-pathway proposed by the X-ray structural analyses given above.

The bacterial CcOs also have an H-pathway analogous to that of the bovine CcO, although some differences are likely to be critical. For example, Asp51 is conserved only in animal CcOs. Arg38Met mutation for a bacterial CcO showed no significant influence on the proton pumping efficiency (Jasaitis, et al., 2001). Met51 side chain smaller than that of Arg would not break the hydrogen-bond network of the H-pathway. The formyl group of heme *a* would interact with the hydrogen-bond network located nearby to trigger pumping proton transfer. Tyr371Phe mutant as active as the wild type (Lee, et al., 2000) would introduce a fixed water at the position of Tyr371-OH group, retaining the hydrogen-bond network. Several residues of the water channel were replaced with less bulky amino acids without any significant influence on the enzyme function. These mutations are unlikely to influence movement of water molecules inside the water channel. These mutational analyses for the bacterial H-pathway mutation analyses, reported thus far, are not sufficient for disproving the proton-pump function of the H-pathway (Shimokata, et al., 2007). In general, only if a mutation of a residue has a clear effect on the function of the protein, it is possible to conclude that this residue has a critical role for the enzyme function.

### 2.5 Diversity in the proton-pumping mechanism of cytochrome *c* oxidase

Proton affinities of the key residues of the D- and K-pathways and their putative proton-exit pathways were theoretically calculated for the three A-family CcOs from bovine and two bacteria, revealing a remarkable similarity in the proton affinity of each residue, consistent to the similarity in the X-ray structures of these CcOs. Based on the simulation results, it has been proposed that the proton pumping mechanism of thee CcOs are identical with each other (Popovic, et al., 2010). On the other hand, the structure of the H-pathway is not completely conserved within the A-family CcOs. For example, Asp51, located at the exit of the pathway, is conserved only in animal CcOs, while plant and bacterial A-family CcOs do not have Asp at the corresponding site, although each of them has a possible proton conducting pathway analogous to the animal H-pathway (Yoshikawa and Shimada, 2015). One of the possible interpretations for this diversity in the H-pathway is that the H-pathway is not involved in the proton pumping function of this enzyme, since such an important function as the proton-pumping must not have any evolutional diversity. However, it is also possible that the diversity in the structure of the H-pathway in the A-family CcOs is induced by evolutional adaptation for differences in the energy requirement, giving a diversity in the proton pump mechanism. Furthermore, different protein structures could perform an identical function. At present, no conclusive experimental evidence has been reported for disproving any of these possibilities.

## 3 Recent progress in the experimental investigation of the mechanism of CcO

### 3.1 Structural findings obtained by improvement of the resolution of the X-ray structure

Resolution of the X-ray structure of bovine heart CcO was improved from 1.8/1.9 Å to 1.5/1.6 Å for the resting oxidized/fully reduced forms, respectively (Yano, et al., 2016). This improvement in resolution corresponds to a 1.73/1.67 times increase in the structural information contained in the X-ray structure. Meanwhile, the X-ray structure of a cytochrome *c*-CcO complex (Shimada et al., 2017b) has set a mile stone for the electron transfer mechanism study between cytochrome *c* and CcO, showing a new protein-protein interaction designated as “soft and specific.” The resolution of the X-ray crystallographic structure of the resting oxidized form has been improved up to a 1.3 Å resolution (Shinzawa-Ito, et al., 2021).

#### 3.1.1 A Mg-containing water cluster

**FIGURE 6:**
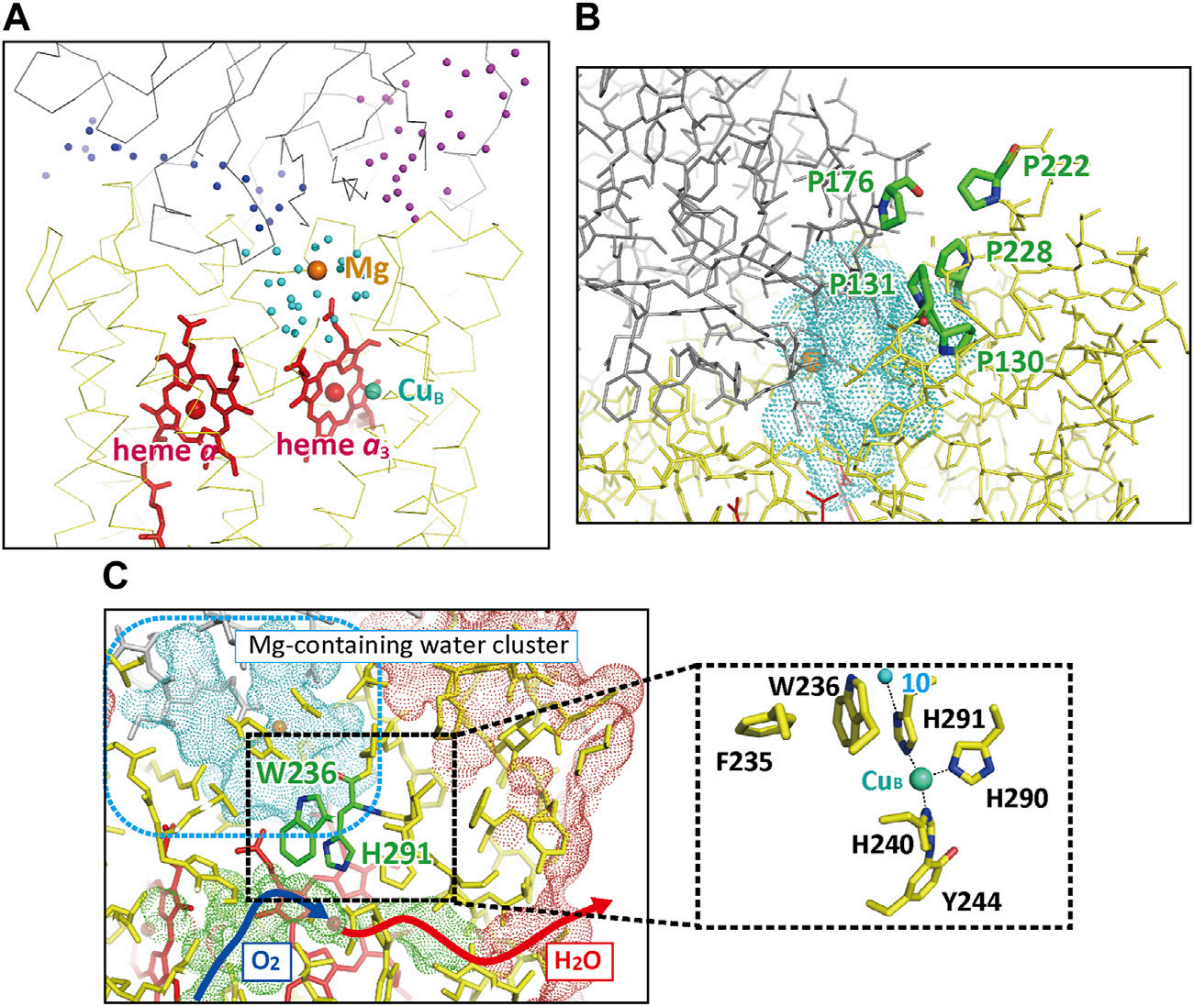
The X-ray structures of the water clusters and the O_2_ pathway. **(A)** The relative location of the three clusters. The positions of the water molecules in each cluster are shown as color-coded spheres, dark blue, light blue (the Mg-H_2_O cluster), and purple. The red and beige spheres indicate the positions of Fe and Mg ions, respectively. **(B)** The proline cluster blocking exchange of water molecules between the protein exterior and the Mg-H_2_O cluster, the water-accessible surface of which is shown by a blue dotted surface. **(C)** The location of the O_2_ pathway closest to the Mg-H_2_O cluster. The green dotted surface indicates the water-accessible surface of the O_2_ pathway. The dark blue and red arrows indicate the possible pathways for O_2_ molecules and the product water molecules. The red, beige, and green spheres indicate the positions of Fe_*a*3_^3+^, the Mg^2+^ ion, and the Cu_B_^2+^, respectively. The red and blue dotted surfaces indicate the molecular surface of CcO in the transmembrane region and the water-accessible surface in the Mg-H_2_O cluster, respectively. The approximate location of the Mg-H_2_O cluster is marked by a blue broken line square. The inset shows structures contributing to the stability of the imidazole group of His291 located in the area marked by a dotted square. The small green sphere denotes a water molecule hydrogen-bonded to His291 (Water 10) located in the Mg-H_2_O cluster. Reprinted with permission from (Yano, et al., 2016).

The X-ray structure of the resting oxidized form showed three large water cluster near the P-side surface as shown in Figure 6A (Yano, et al., 2016). The structures of the interfaces between these clusters indicate that any proton exchange is impossible between these clusters. The water cluster closest to heme *a*_3_, the blue-colored cluster in Figure 6A, containing Mg^2+^ ion site, is designated as Mg-H_2_O cluster. The cluster has a channel-like structure extending to the P-side surface as shown in Figure 6B. However, a 1.0 Å probe analysis shows that direct contact is impossible between the cluster and the P-side phase. Furthermore, five proline residues surround the channel to stiffen the channel (Figure 6B). The pathway for the O_2_ inlet from the transmembrane surface of the subunit III and the H_2_O (the CcO reaction product) outlet are located near the Mg-H_2_O cluster as marked by the dark blue and red arrows in Figure 6C. The junction point is at heme *a*_3_ of the O_2_ reduction site. The two propionate groups of heme *a*_3_ interact with the Mg-H_2_O cluster by forming hydrogen bond with the water molecules in the cluster. However, the -CH_2_-CH_2_- moiety and the surrounding amino acid residues seem to block any proton exchange between the water cluster and the mobile waters in the O_2_/H_2_O pathway. One of the three imidazole groups coordinated to Cu_B_ is hydrogen-bonded to a fixed water (water 10) in the water cluster (Figure 6C inset).

**FIGURE 7:**
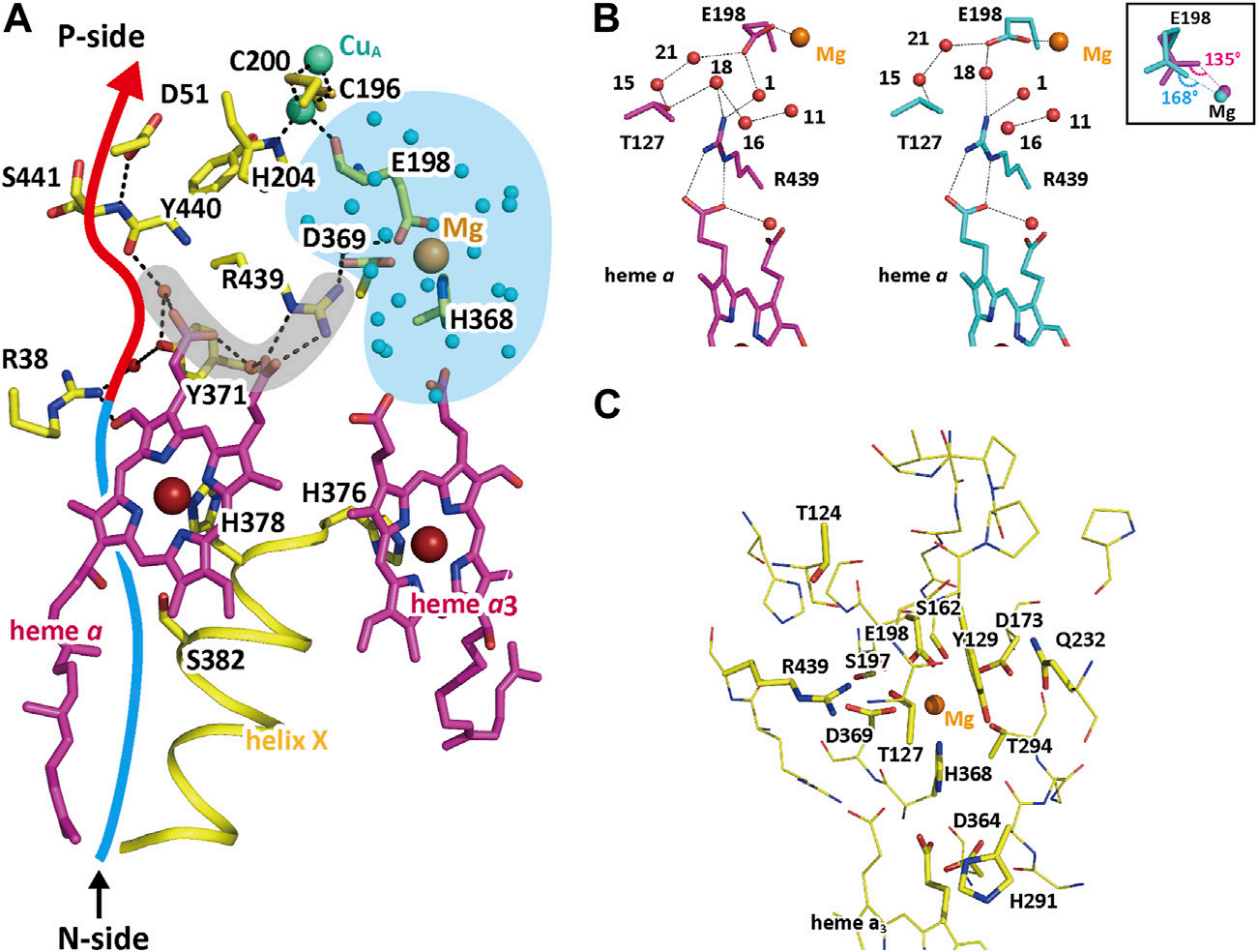
The structure of the short hydrogen-bond network and the redox-coupled conformational changes in the Mg-H_2_O cluster. **(A)** The structure of the short hydrogen-bond network (marked by a gray area) connecting the Mg-H_2_O cluster (shown by a blue area) with the hydrogen bond network (marked by a red arrow) of the H-pathway. To improve clarity, only the hydrogen-bonds in the short hydrogen bond network and the hydrogen-bond network of the H-pathway are shown with broken lines. The structure of the Mg-site is the structure adopted when the enzyme is in the oxidized state. The red and blue arrow denotes the location of the hydrogen-bond network and the water channel of H-pathway, respectively. **(B)** The redox-coupled conformational changes in the Mg site. The purple and blue structures indicate those in the oxidized and reduced states, respectively. To preserve clarity, only the water molecules (orange spheres) that are associated with the conformational changes are shown. The dotted lines indicate hydrogen bonds. The redox-coupled coordination-structural change occurring at the E198 carboxyl group is shown in the inset. The carboxyl group of Glu198 interacts with the guanidino group of Arg439 via water molecules, giving a possible proton transfer pathway to the short hydrogen bond network. The redox coupled structural changes in coordination of the carboxyl group of Glu198 to the Mg^2+^ (Figure 7B inset) induces a redox-coupled structural changes in the hydrogen bond structure between Glu198 and Arg439, suggesting significant changes in proton transfer efficiency between Glu198 and Arg439 at the one end of the short hydrogen bond network. **(C)** The location of hydrophilic functional groups included in the Mg-H_2_O cluster. To preserve clarity, water molecules detectable in the cluster are not shown. The thin sticks indicate non-polar amino acid residues interacting with their main chain groups and polar groups which are unlikely to accept protons reversibly. Tyr129, Arg173, Asp364, His368, His291, Arg438, and the two propionate groups of heme a_3_ are able to accept protons reversibly. The polar but non-charged residues, Ser162, Ser197, Thr124, Thr127, Thr294, and Gln232, increase the effective dielectric constant of the interior of the Mg-H_2_O cluster. Reprinted with permission from (Yano, et al., 2016).

The Mg-H_2_O cluster, colored in blue in Figure 7A, is connected to the H-pathway (the blue and red arrow) with a short hydrogen bond network shaded in gray in Figure 7A (Yano, et al., 2016). The Mg^2+^ in the cluster is coordinated to the carboxyl group of Glu198, which is coordinated to the Cu_A_ site with the peptide C=O group (Figure 7A). Glu198 shows a redox-coupled structural changes as given in Figure 7B, suggesting significant changes in proton transfer efficiency between Glu198 and Arg439 at the one end of the short hydrogen bond network.

The improved X-ray structures of the Mg-H_2_O cluster in both oxidation states (Yano et al., 2016) provide well-resolved water molecules, giving essentially identical numbers of the water molecules, 20.75 and 20.30, for the resting oxidized and fully reduced forms, respectively. This result suggests absence of water molecule exchange with those located outside of the cluster, consistent with the water-accessibility analyses of the cluster. Many hydrophilic amino acid residues and the two heme *a*_3_ propionates are included in the Mg-H_2_O cluster as indicated in Figure 7C. Six amino acid residues and the two propionate groups of heme *a*_3_ in the figure are able to accept protons reversibly. The 20–21 water molecules tightly packed in the interior of the Mg-H_2_O cluster, many polar but non-charged residues, and the peptide groups tightly hydrogen-bonded with each other, as shown in Figure 7C, are likely to provide a dielectric environment of the interior of the Mg-H_2_O cluster closely similar to that of the N-side phase. The above structural findings indicate the sufficient capacity of storage of, at least, four protons in the Mg-H_2_O cluster. Furthermore, the redox-coupled structural changes in Glu198 would facilitate the reversible and redox-coupled proton accepting capacity between the cluster and the N-side phase.

#### 3.1.2 The pathway for the substrate (O_2_) inlet and the product (water) outlet

The outlet pathway for the product water was identified as described in Figure 6C. Many polarized oxygen atoms surround the pathway giving a highly hydrophilic environment. All residues surrounding the O_2_-inlet pathway are hydrophobic except for Glu242. The location of the opening of the product (water) exit at the transmembrane region, as shown in Figure 6C, is critical for effective suppression of proton back leak from the P-side when the product water molecules are released. As described above, the inside of the O_2_ inlet is highly hydrophobic while that of the H_2_O outlet is highly hydrophilic. The D-pathway is attached to the O_2_ inlet pathway at Glu242. Thus, protons, transferred from the D-pathway to the O_2_ inlet pathway, would be readily transferred to the H_2_O outlet pathway, unless the O_2_-reduction site traps them for water formation. The D-pathway mechanism proposes that pumping protons are transferred through the D-pathway to the PLS located near the BNC. Then, the H_2_O outlet pathway would readily take up the pumping protons and release them from the H_2_O outlet, located in the trans-membrane surface, to dissipate the membrane potential. Therefore, this structure does not support the D-pathway mechanism.

### 3.2 Determination of the X-ray structures of intermediate forms

#### 3.2.1 Structures of the intermediate forms

X-ray structural changes of the fully reduced CcO in crystals after exposure to excess amounts of O_2_ were followed by static X-ray diffraction experiments in SPring-8, for determination of the intermediate forms (Shimada, et al., 2021). The data sets were analyzed by multiple structural analyses (Shimada, et al., 2021). The multiple structural analysis is indispensable, since data sets obtained from the CcO crystals during the CcO reaction are highly likely to include multiple intermediate forms. If the X-ray diffraction data were analyzed without taking the existence of the multiple components into account, the analysis would provide an unreal structure, corresponding to the weighted average of multiple component structures.

**FIGURE 8:**
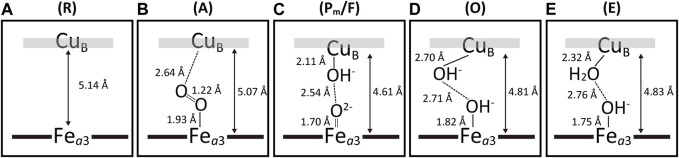
Schematic representations of the X-ray structures of the intermediate forms. Panels **(A–E)** show those of the R-, A- Pm/F-, O- and E-forms, respectively. These structures were obtained from the final atomic models determined by the X-ray structural analyses for the fully reduced bovine heart CcO crystals exposed to excess O_2_ for different period. The structural analyses were performed taking the possibility of multiple structures into account. The accuracy of the determined structures depends on the occupancy of each intermediate forms. Because of the low occupancy of the A-form (∼20%), the reliability of these structural results is significantly lower than those of the other forms. However, the difference in the atomic distances between Cu_B_-O and Fe_*a*3_-O in panel B is significant. The structure given in panel **(C)** was determined from the crystals of a 1:1 p_m_/F mixture. The structural difference between the two forms is too small to detect at the resolution of the X-ray diffraction experiments. Reprinted after some modifications with permission from (Shimada, et al., 2020; Shimada, et al., 2021).

Figure 8 shows schematic representations of the BNC of the intermediate forms, R, A, P/F mixture, O and E. As shown in panel A**,** the R form has no ligand in both metal sites. The A form shows an oxymyoglobin-type structure in panel B. The structure suggests that the Cu_B_-O distance is significantly longer than the Fe_*a*3_-O distance (2.64 Å vs. 1.93 Å) (Shimada, et al., 2021). Panel C shows a schematic representation of the O_2_-reduction site of the X-ray structure obtained from 1:1 P_m_/F mixture crystals. The difference in the structures between the two forms is too small to detect at the resolution. The Fe_*a*3_-O distance of 1.70 Å is consistent with the bond length of Fe^4+^ = O^2−^, not that of Fe^4+^-OH^−^. The distance between the two oxygen atoms of the ligands (2.54 Å) indicate the existence of a short hydrogen bond between the two ligands (Shimada, et al., 2020). In the structure of the BNC of the O-form shown in panel D, the Fe_*a*3_-O distance of 1.82 Å is consistent with that of Fe^3+^-OH^−^, while the unusually long Cu_B_-O distance, 2.70 Å, was obtained. The BNC structure of the E-form in panel D shows significantly shorter Cu_B_-O distance compared with that of the O-form (2.32 Å vs. 2.70 Å). No significant structural difference was detectable between the O- and E-forms except for the Cu_B_-O distance, as shown in panels D and E. The Cu_B_-O distance, 2.32 Å, suggests that the Cu_B_ is in either Cu^1+^-OH^−^ or Cu^1+^-OH_2_ state (Shimada, et al., 2021). The O→E transition is coupled with uptake of a proton from N-side to the O_2_ reduction site. Thus, Cu_B_ in the E-form is likely to accept the proton to form Cu^1+^-OH_2_. Protonation of Cu^1+^-OH^−^ would stabilizes the Cu^1+^ state by the net positive charge increase. The water channel of the H-pathway is in the closed state in all the intermediate forms except for the R form, which has the open state (Shimada, et al., 2020; Shimada, et al., 2021).

A cryo-EM analysis for the intermediate forms of a bacterial CcO has been reported (Kolbe, et al., 2021) providing amazing structural findings, such as a peroxide dianion, intact dioxygen molecule and a superoxide anion bound at the O_2_-reduction site of the O, P and F forms, respectively. The reported structure indicates that the Fe_*a*3_-Cu_B_ distance of the R-form is as short as 3.7 Å. The short distance suggests a strong electromagnetic interaction between the two metal ions, while no significant anomality in the absorption spectrum is detectable in the report. However, conditions for the experiments and the structural analyses of image data have not been provided sufficiently for evaluation of their final structural results.

#### 3.2.2 Significance of the X-ray structural findings of the intermediate forms

Based on the X-ray structure of the CO-bound fully reduced CcO, it has been proposed that uneven bridging of O_2_ to the two metals in the BNC is critical for slow formation of the peroxide-bound form so that the steady state level of the peroxide intermediate is lowered down to the negligible level (Yoshikawa and Shimada, 2015). The result summarized in Figure 8B for the A-form confirms experimentally the proposal.

It has been proposed, by comparison of the X-ray structures of various inhibitor-bound forms, that the water channel of the H-pathway opens only in the R-form in the catalytic cycle (Yoshikawa and Shimada, 2015). However, it is possible that the water channel is open also in the intermediate forms. This long-standing proposal has been confirmed experimentally by these X-ray structural analyses of the intermediate forms as given above (Shimada, et al., 2020; Shimada, et al., 2021).

For maximal efficiency in proton/electron coupling, each of the four transitions coupled with a proton pump in the catalytic cycle of CcO should be essentially irreversible. In other words, the intermediate forms, P_m_, F, O and E, should have high electron affinity sites. The X-ray structural analyses for the intermediate forms as given in the previous section provided various insights in this point as follows: The unusually long Cu_B_-O distance in the O-form suggests very weak negative charge influence of the OH^−^ to Cu_B_^2+^, indicating that Cu_B_^2+^ has an extremely high electron affinity (Shimada, et al., 2021). The X-ray structures of the F-form (as well as the P_m_ form) shows the Fe_*a*3_^4+^ = O^2−^ structure, as a high electron affinity site, confirming crystallographically the structure of the F-form, proposed by a resonance Raman analysis as shown in Figure 1. The high electron affinity of Cu_B_ in the E-form as described above is likely to indirectly increase the effective electron affinity of the Fe_a3_ site by decreasing the electron population in Fe_*a*3_. The significant acceleration of the electron transfer to the Fe_*a*3_ site by reduction of Cu_B_^2+^ was found by a rapid freeze EPR and stopped flow absorption spectral analyses (Jancura, et al., 2006), confirming kinetically that the Fe_*a*3_^3+^ site in the E-form has high electron affinity. However, the structure of Fe_*a*3_ itself does not suggest high electron affinity. Although Tyr244 neutral radical has been proposed as the high affinity site of the P_m_-form, X-ray structural identification of the radical structure would be impossible even at the highest resolution presently available (1.3 Å) (Shinzawa-Itoh, et al., 2021). This proposal has been confirmed spectroscopically as described below.

#### 3.2.3 The proton pool

The improved X-ray structures of bovine CcO strongly suggest that the Mg-H_2_O cluster can accept at least four protons reversibly (Yano et al., 2016) as mentioned in Section 3.1.1. Thus, four protons for each catalytic cycle are collected into the Mg-H_2_O cluster from the N-side in the open state (in the R-form) and pumped to the P-side from the Mg-H_2_O cluster in the closed state, one by one, driven by heme *a* oxidation. Thus, in each of the four proton-pumping transitions (P→F, F→O, O→E, and E→R), one proton is released to the P-side coupled with uptake of one water-forming proton from the N-side. However, this is inconsistent with an experimental finding that, in each of the two transitions, P→F and F→O, two proton equivalents are collected from the N-side coupled with release of one proton to the P-side as described above, suggesting that one pumping proton in addition to one water-forming proton is collected from the N-side in each proton-pumping transition (Faxén, et al., 2005). This collected proton is unlikely to be transferred to the Mg-H_2_O cluster instantaneously in each proton-pumping transition since the water channel is closed. Thus, a pool for the collected proton is necessary below the open/closed point of the water channel. In each proton pumping transition, one proton is taken up from the N-side to the proton pool. Thus, the four protons kept in the proton pool in each catalytic cycle are transferred to the Mg-H_2_O cluster in the R-form. The proton pool are likely to facilitate effective proton collection from the N-side phase under extremely low proton concentration. An experimental result supports the existence of the proton pool (Zaslavsky et al., 2004). Further details are given in Supplementary Materials for the reference (Shimada et al., 2017) and in the reference (Wikström et al., 2018).

### 3.3 Spectroscopic studies on the intermediate forms

#### 3.3.1 Identification of an intermediate form between the A and P forms

It has been well-established that, in both P_m_ and P_r_ forms, O_2_ bound at the A-form is completely reduced to give Fe_*a*3_^4+^ = O^2−^, and Cu_B_^2−^-OH^−^. Thus, no intermediate species during the O_2_ reduction process during CcO reaction was identified experimentally, although formation of a peroxide-type intermediate has been expected by various synthetic-chemical analyses (Yoshikawa and Shimada, 2015). Perhaps, the first successful experimental identification of an intermediate between the A and P_m_ forms was accomplished by a flash photolysis analysis for the O_2_ reduction by the fully reduced *Thermus thermophilus* CcO in which heme *a* is replaced with heme *b* (Poiana, et al., 2017). At 10°C and pH 7, after photolysis of the CO-bound *ba*_3_ CcO, absorbance changes at 610 nm showed O_2_-binding at a time constant of 3.6 µs, followed by P_r_ formation at 110 µs, while at 560 nm, heme *b* oxidation was observed at 11 µs. This result indicates that the heme *b* oxidation is 10 times faster than P_r_ appearance. With an increase in pH up to 10, the heme *b* oxidation was slowed down to a time constant of 38 μs (3.5 times slowed) without any significant pH effect on the O_2_ binding and on the P_r_ formation. As described above, in bovine and bacterial *aa*_3_ CcO, P_r_ is formed by electron donation from reduced heme *a* (Fe_a_^2+^) to the A-form as follows,

$$A\left({Fe}_{a3}^{2+}-O_{2},{Cu}_{B}^{1+},Tyr244OH\right)+{Fe}_{a}^{2+}\to \mathrm{Pr}\left({Fe}_{a3}^{4+}=O^{2+},{Cu}_{B}^{2+}-{OH}^{-},Tyr244O^{-}\right)+{Fe}_{a}^{3+},$$

where Tyr244O^−^ denotes deprotonated Tyr244. The above result for the *ba*_3_ CcO, that is, the faster heme *b* oxidation than the Pr appearance indicates that the Fe_*b*_^2+^ oxidation provides an intermediate form before the Pr formation. The pH sensitivity of the heme *b* oxidation suggests that the new intermediate is protonated. Thus, a hydroperoxo-bound form, Fe_*a*3_^3+^-O-OH^−^, has been proposed for the new intermediate form, including Cu_B_^1+^and Tyr244O^−^, assuming that Fe_*a*3_^2+^-O_2_ has absorption spectrum closely similar to that of Fe_*a*3_^3+^-O-OH^−^ at least at 610 nm, since no significant spectral change is detectable corresponding to the heme *b* oxidation at 610 nm (Poiana, et al., 2017). However, the assumption is in conflict with a consensus in the field of the hemoprotein chemistry. That is, upon the transition from Fe^2+^-O_2_ (or Fe^3+^-O_2_^−^) to Fe^3+^-O-OH^−^, the hemochrome type spectrum in the α-band region (550–600 nm) is eliminated giving significant absorbance decrease in the region. The consensus has been confirmed recently by a synthetic chemical approach (Kim, et al., 2020). Extensive spectroscopic (especially resonance Raman) analyses are desirable for identification of the structure of the intermediate.

#### 3.3.2 The existence of a neutral radical of Tyr244 in the P_m_ form

**FIGURE 9:**
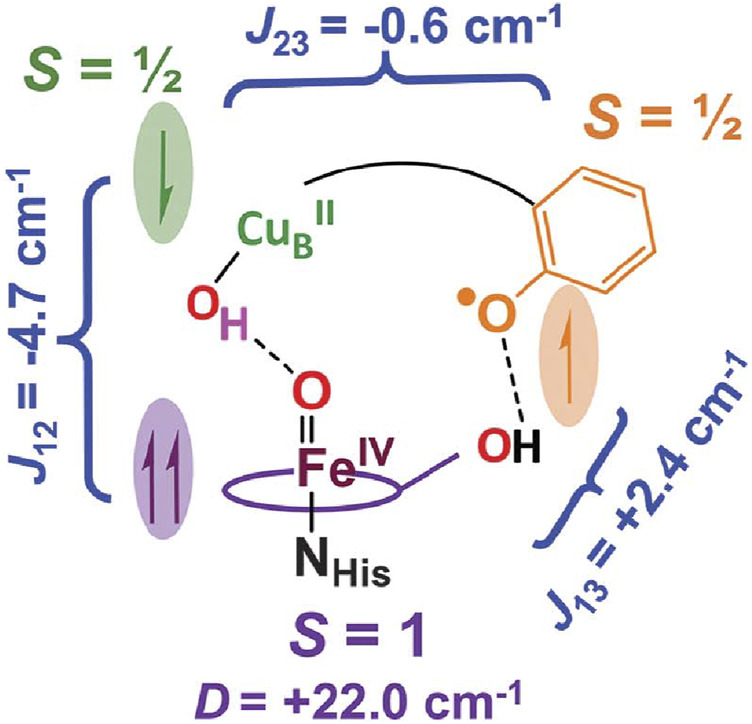
A schematic representation of the magnetic couplings in P_m_, discovered by VTVH MCD analysis of the P_m_ state of *E. coli* ubiquinol oxidase. One of three imidazole groups of histidines, His240, coordinated to Cu_B_ and cross-linked to Tyr244 phenol group colored in beige, is schematically shown by a black curve. Dotted lines denote hydrogen bonds. Reprinted with permission from (Jose, et al., 2021).

As mentioned above, in the P_m_ form, the bound O_2_ has been completely reduced to provide Fe_*a*3_^4+^ = O^2−^ and Cu_B_^2+^-OH^−^. The X-ray structure of the BNC strongly suggests that one of the four electron equivalents and one proton equivalent required for formation of the P_m_ form are from Tyr244OH, leaving a neutral radical (Tyr244O•). However, in spite of various experimental trials (Jose, et al., 2021 and references therein), the existence of the radical in the P_m_ form had not been experimentally proven. The Fe^4+^ = O^2−^ site in the P_m_ form was examined extensively by variable-temperature, variable-field magnetic circular dichroism (VTVH-MCD) spectroscopy using *E. coli* ubiquinol oxidase which has a ubiquinol site instead of Cu_A_ site, which obscures the MCD feature of the Fe^4+^ = O^2−^ site (Jose, et al., 2021). The VTVH-MCD results showed that the Fe_*O*_^4+^ = O^2−^ is magnetically coupled to additional paramagnetic sites assignable to the Cu_B_^2+^ and Tyr244O• sites, as schematically shown in Figure 9. The antiferromagnetic coupling between the Fe_*O*_^4+^ = O^2−^ and Cu_B_^2+^-OH^−^ is induced by the super-exchange pathway through the hydrogen bond between the two oxygen atoms indicated by a dotted line in the Figure. The antiferromagnetic coupling between the Cu_B_^2+^ and Tyr244O• is facilitated by His240 which is covalently linked to Tyr244 and coordinated to Cu_B_. The role of His240 is described by a black curve for the sake of simplicity. The ferromagnetic coupling between Tyr244O• and the Fe_*O*_^4+^ = O^2−^ is promoted by the hydrogen bond between the OH group of the hydroxyfarnesyl ethyl group and O• group of Tyr244. These magnetic couplings and spin density values prove that the neutral radical in P_m_ is located at Tyr244 (Jose, et al., 2021). The neutral radical is reduced to TyrO^−^ in the Pm→Pr transition (Jose, et al., 2021).

#### 3.3.3 Role of F_r_-form, an intermediate between the F- and O-forms, in the respiratory control mechanism

**FIGURE 10:**
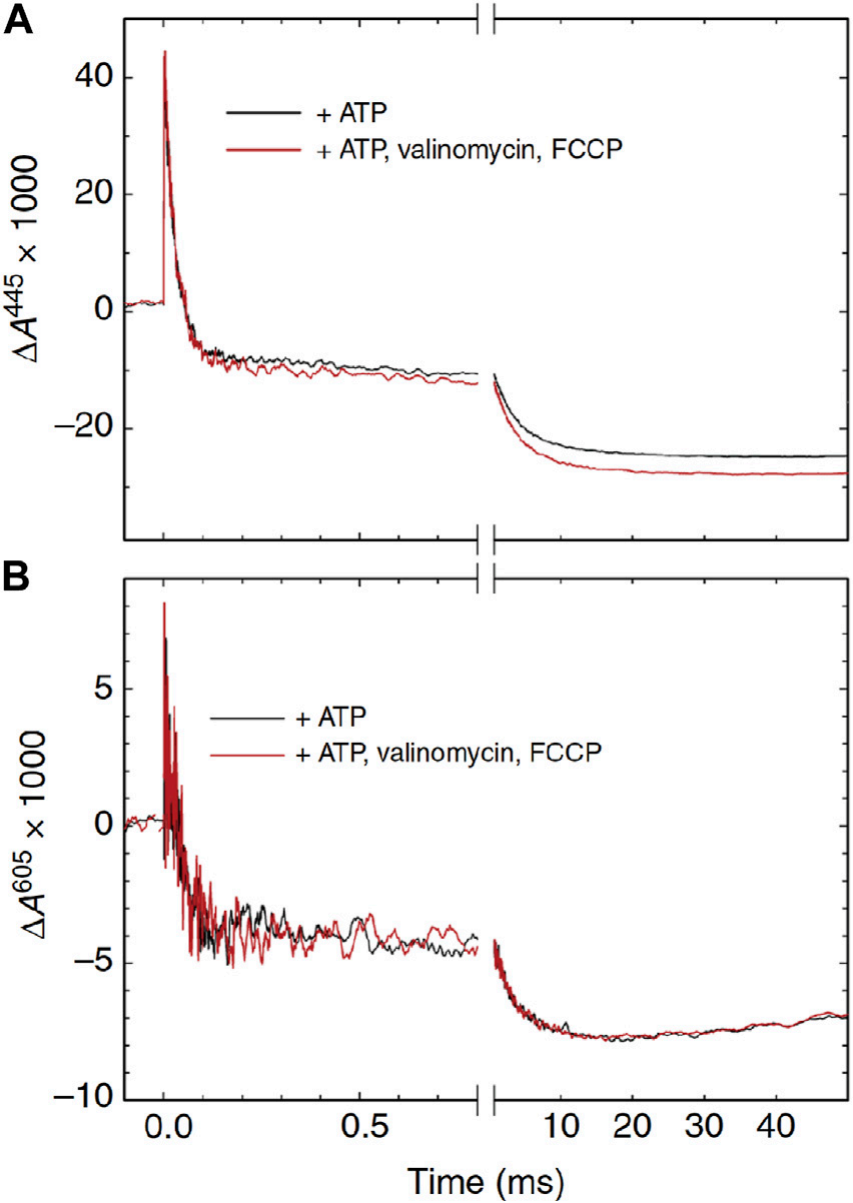
Absorbance changes associated with the reaction of CcO with O_2_ in submitochondrial particles. The CO-bound fully reduced CcO was mixed with an O_2_-saturated solution containing ATP. After 0.8 s, the reaction of CcO with O_2_ was initiated by a laser flash. At 445 nm [panel **(A)**], the absorbance change reflects the redox states of both hemes *a* and *a*_3_, while at 605 nm, it does mainly that of heme *a* [panel **(B)**]. Reprinted with permission from (Bjorck and Brzezinski, 2018).

**FIGURE 11:**
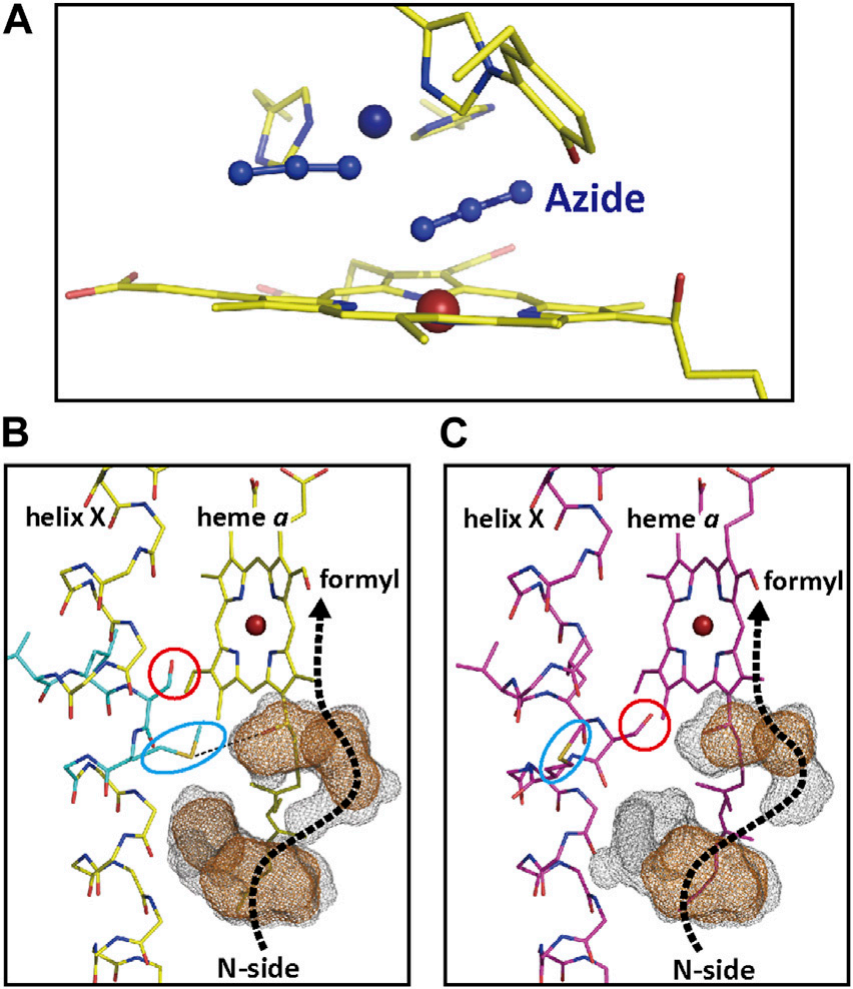
The structures of the two-azide bound form of CcO. **(A)** The atomic model of the O_2_-reduction site of the two-azide bound form. The dark blue and brown spheres denote the positions of Cu_B_^2+^ and Fe_*a*3_^3+^. **(B)** The structure of the water channel of the H-pathway of the two azide bound form near its upper end close to the heme *a* formyl group. The brown and gray dotted surfaces denote water cavities determined using probes with a radius of 1.2 and 0.8 Å, respectively. A possible water pathway to the formyl group from the N-side is marked by a dotted thick arrow. Side chains of Ser382 and Met383 are located as shown by a red circle and a blue oval, respectively. **(C)** The structures of the water channel in the resting oxidized form structures. Reprinted with permission from (Shimada, et al., 2018).

It has long been known that mitochondrial respiration is controlled by the proton motive force (pmf). However, its mechanism is still essentially unknown. The effect of the pmf on the CcO function was examined by using an artificial vesicle preparation from the mitochondrial inner membrane, designated as SMP (submitochondrial membrane particle), which is a preparation of vesicles inside-out relative to intact mitochondria (Bjorck, and Brzezinski, 2018). By addition of ATP to the outside of SMPs, the pH in the inside of the vesicle can be lowered to create the pmf to the SMP membrane. The effects of the pmf on the oxidative phase of the CcO reaction were examined by following the absorption spectral changes of the CcO in the SMPs after flash-photolysis of the CO-bound fully reduced CcO at 445 nm and 605 nm in the presence of ATP (Figure 10). Both hemes *a* and *a*_3_ contribute to the absorbance changes at 445 nm roughly equally, while the 605 nm changes are mainly induced by heme *a*. Following large changes due to the R→A and A→P_r_ transitions coupled with heme *a* oxidation within the initial 100 μs and a much smaller and slower change for about 1 ms, due to P_r_→F, without a significant redox state change in heme *a*, the F→O transition with the time constant of ∼5 ms coupled with heme *a* oxidation was observed. The effects of the pmf were examined by the addition of valinomycin and FCCP, which collapse completely the pmf (the red traces). The red traces at 605 nm are essentially identical to those of the black traces (Figure 11B), indicating that heme *a* oxidation is insensitive to the membrane potential. However, significant decrease (14%) in the amplitude of the 5 ms phase without changing the time course is detectable at 445 nm (Figure 10A).

Absorbance spectrometric and electrometric analyses previously suggested that during the F→O transition, F receives one electron equivalent without significant influence on its absorbance spectrum giving an intermediate form, Fe_*a*3_^4+^ = O^2−^, Cu_B_^1+^, designated as Fr and that only one of the two transitions, F→F_r_ and F_r_→O, is coupled with a proton-pump. However, because the F_r_ formation is much slower than the F_r_→O transition, the F_r_ is not detectable in the F→O transition (Zaslavsky, et al., 1993; Zaslavsky, 1999; Brändén, et al., 2006).

The third phase at 445 nm in the presence of the pmf (the black trace) corresponds to the absorbance decrease only due to oxidation of heme *a* (Figure 10A). In other words, the F→O transition is suppressed by the pmf. In fact, the decrease in the amplitude of the 5 ms process upon application of the pmf is essentially identical to the absorbance difference between the F- and O-forms at 445 nm. However, heme *a* oxidation was insensitive to the pmf, as shown by the 605 nm traces in Figure 10B. This result indicates that heme *a* oxidation induces the transition from the F-form to the F_r_-form, which has the absorbance spectrum identical to the F-form. Perhaps, this is the first conclusive experimental evidence for the involvement of the F_r_-form in the normal catalytic process. The F→Fr transition is insensitive to the membrane potential, since this transition is driven by the electron transfer from heme *a*, which is parallel to the membrane surface. However, the Fr→O transition, which is coupled with proton uptake from N-side and proton pumping, is suppressed by the pmf. Thus, these results strongly suggest that the activity of CcO under turnover conditions is controlled at the Fr→O transition, which produces the initial intermediate of the catalytic cycle of the CcO reaction (i.e., the O-form). Thus, this site is reasonable for the feedback regulation of CcO function. Important future works would be experimental trials for identification of the structural basis for avoiding the membrane potential influence on the Pr-F transition.

### 3.4 Experimental studies on mechanism of the proton pump

#### 3.4.1 The structure of the water channel of the H-pathway

Atomic molecular dynamics simulations (Sharma et al., 2017) indicated that protonation of His413 in the water channel of the H-pathway was necessary for incorporation of water molecules into the water channel to form a proton transfer pathway from the N-side to the hydrogen bond network of the H-pathway. The X-ray structure at 1.8 Å resolution (PDB ID: 1V54) shows that the main part of the water channel is not sufficiently hydrated for proton transfer through it, indicating that proton-transfer through the water channel is impossible. However, the improved X-ray structures at 1.65 Å or better (PDB ID: 5B1A, 5B1B, 5ZCQ, 5ZCP, 7COH, 7VUW, 7VVR, and 7YPY) show that the water channel is fully hydrated to provide the structure identical to that predicted by the above simulations, assuming that His413 is protonated. Thus, these improved X-ray structures demonstrate that the proton transfer through the water channel is possible. It seems that the 1.8 Å resolution (PDB ID: 1V54) is not sufficiently high for resolving these water molecules. This point has been mentioned previously (Shimada, et al., 2018).

#### 3.4.2 Mutation analysis of the H-pathway of the yeast, *Saccharomyces cerevisiae*

Functions of the K-, D-, and H-pathways of a mitochondrial CcO from the yeast, *Saccharomyces cerevisiae*, were examined by a recently developed method for mitochondrial DNA mutagenesis (Maréchal, et al., 2020). The mutants prepared include Asn99Asp, Glu243Asp, and Ile67Asn in the D-pathway, Gln411Leu, Gln413Leu, Ser382Ala, Ser458Ala, Ser455Ala, and Ser52Asp in the H-pathway, and Thr316Lys in the K-pathway (The yeast numbering is used in this section.) The effects of these mutations were examined by the cell growth rates, the oxygen consumption rates of the mitochondrial membrane fragments, and the energy coupling efficiency (H^+^/e^−^ ratio) in intact isolated mitochondria respiring a respiratory substrate, α-ketoglutarate. The H^+^/e^−^ ratio was estimated from the ADP/O ratios in state 3 subtracting the background O_2_ consumption in state 4. No significant effect of these H-pathway mutations is detectable on these CcO activities. Furthermore, the kinetics of electron and proton transfer during the reaction of the fully reduced forms of the Ser382Ala and Ser458Ala mutant CcOs with O_2_ were investigated by a flow flash method to show that these mutant CcOs were as active as the wild-type CcO (Bjorck, et al., 2019). Thus, it has been concluded that H-pathway is not involved in the proton pumping function of the *S. cerevisiae* CcO.

However, the structural modifications introduced by the H-pathway mutations, as mentioned above, do not seem to influence significantly the proton pumping function of the H-pathway. The five mutation sites for Gln441, Gln413, Ser382, and Ser458 are located in or near the water channel of the H-pathway through which mobile water molecules transfer protons to the hydrogen bond network starting from Arg38 hydrogen-bonded to the formyl group of heme *a*. In order to block the water accessibility, mutation to the bulkier residue is necessary as in the case of the double mutation for bovine CcO, Val386Leu/Met390Trp (Shimokata et al., 2007). The structural changes in these Gln411Leu, Gln413Leu, Ser458Ala, and Ser455Ala mutations are too small to effectively block the water channel. Ser382 is hydrogen-bonded to the OH group of the hydroxyfarnesyl ethyl group of heme *a* to eliminate a water cavity in the water channel to block the water access to the hydrogen bond network. However, an energy minimization analysis suggests that the structural change in the Ser382Ala mutation is too small to eliminate the closed/open transition as detectable upon complete reduction of the resting oxidized CcO. Ser52Asp mutation, located in a fairly hydrophilic environment in the P-side end of the H-pathway, is highly unlikely to affect the function of the H-pathway. In general, a mutational result that provides no significant effect on its function is not conclusive evidence that the mutated residue has no critical role in its function, as mentioned in Section 2.4. Furthermore, the various structural diversities in the H-pathway of mitochondrial CcO, such as in Asp51, suggest significant diversity in the proton pumping function of the H-pathway. It is possible that the H-pathway pumps protons in cow, but not in yeast, as discussed in Section 2.5.

In the *S. cerevisiae* CcO, His413 in the mammalian CcO is replaced with Gln413. If the H-pathway in the *S. cerevisiae* CcO has a proton-pumping function identical to that of the mammalian CcO, the exchange of His413 to Gln413 suggests that the protonation of His413 is not necessary for the proton transport function of the water channel of the mammalian CcO. High-resolution structural analysis for the yeast CcO is desirable.

#### 3.4.3 Electrostatic interaction between the pump protons on the hydrogen-bond network of the H-pathway and the net positive charges created upon heme *a* oxidation

The H-pathway mechanism proposes that proton pump of CcO is driven by the electrostatic repulsions between the pumping protons on the hydrogen bond network of the H-pathway and the net positive charges created upon oxidation of the heme *a*. In fact, X-ray structure shows that heme *a* is hydrogen-bonded to the hydrogen bond network of the H-pathway (Figure 3). In order to evaluate whether the electrostatic interactions are strong enough for the proton pumping, azide-induced structural perturbations of the H-pathway were examined crystallographyically. The two-azide binding to the O_2_-reduction site of the resting oxidized CcO (Figure 11A) induced significant structural changes in the side chains of Ser382 and Met383, as shown by a red circle and a blue oval, respectively, as described in Figures 11B, C. These side chains are in the water channel of the H-pathway near its upper end close to the heme *a* formyl group, which is at the bottom end of the hydrogen bond network of the H-pathway. The brown and gray dotted surfaces denote water cavities determined using probes with a radius of 1.2 and 0.8 Å, respectively. A possible water pathway to the formyl group from the N-side is marked by a dotted thick arrow in each panel. In the resting oxidized form structure (Figure 11C), although a possible water pathway is drawn by a dotted arrow, water molecules are not accessible to the formyl group, at least, in the physiological time scale. The continuous gray surfaces of the water channel structure of the two azide-bound form (Figure 11B), indicate that water molecules are accessible to the formyl groups within the physiological time scale. On the other hand, it has been reported that, the redox potential of heme *a* was significantly decreased by azide (Wilson, et al., 1972). The water channel opened by the azide binding is highly likely to decrease the proton level in the hydrogen-bond network of the H-pathway. This electron affinity decrease in heme *a* upon azide binding strongly suggests the existence of significant electrostatic interactions between the protons on the hydrogen-bond network of the H-pathway and heme *a*.

#### 3.4.4 Time-resolved X-ray structural analyses for CcO reaction

**FIGURE 12:**
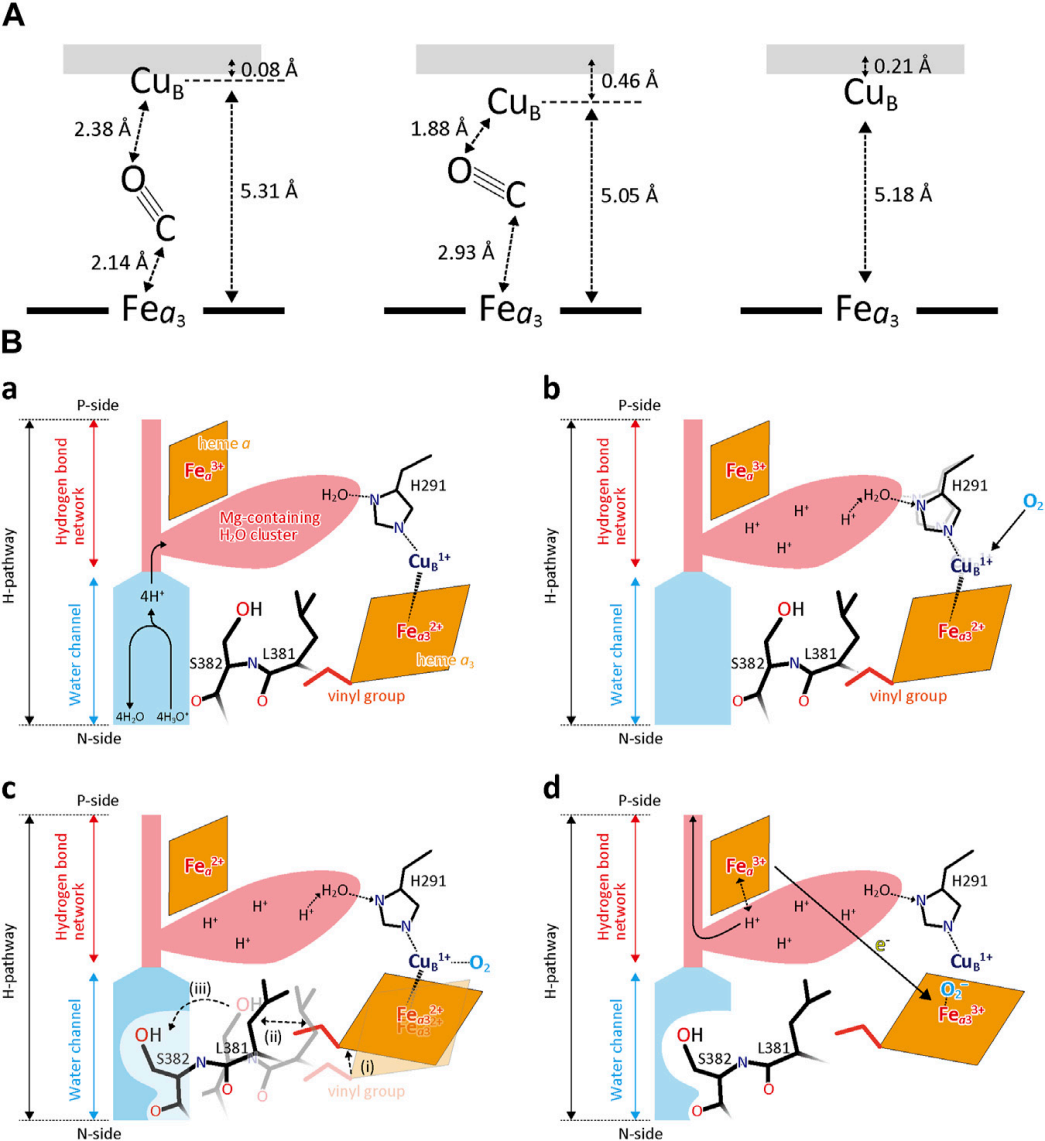
Time-resolved X-ray structural changes associated with CO release from CcO. **(A)** A schematic representation of geometry of CO in the O_2_ reduction site before flash (left), 20 ns(center), and 100 μs (right) after photolysis. **(B)** A schematic representation of a water channel closure mechanism, based on the results shown in **(A)**. **(a)** The O_2_ reduction site is in the fully reduced state under turnover conditions. The Mg^2+^- H_2_O cluster is connected to Cu_B_ via H291 and a fixed water molecule (Water10 in Figure 6C) in the storage site. The heme *a*_3_ vinyl group is in van der Waals contact with Leu381. Protons are transferred by H_3_O^+^ through the open water channel. **(b)** The increase in the O_2_-affinity of Cu_B_^1+^ caused by distortion of the regular trigonal coordination of Cu_B_^1+^ which is induced by protonation of the fixed water upon full protonation of the Mg-H_2_O cluster. **(c)** Closure of the water channel upon O_2_ binding to Cu_B_ as described in the dotted lines. **(d)** Proton pump after the channel closure. After migration of O_2_ to Fe_*a*3_^2+^, Fe_*a*_^2+^ is oxidized to pump protons. Reprinted with permission from (Shimada, et al., 2017a).

It has been shown that CO binding to the fully reduced form induces the open to closed transition of the water channel essentially identical to that detectable upon complete oxidation of the fully reduced form (Yano, et al., 2016). In order to investigate the dynamic aspects of the closed/open structural transition in the water channel of the H-pathway, the structural changes after photolysis of the CO-bound fully reduced CcO were examined by using time-resolved XFEL and infrared (IR) techniques with a pump-probe method (Shimada, et al., 2017b). Figure 12 illustrates schematically the structures of the BNC before CO-photolysis and 20 ns and 100 μs after CO-photolysis. At 20 ns, CO was migrated stoichiometrically to Cu_B_^1+^, concomitantly with a decrease in the Fe_*a*3_-Cu_B_ distance from 5.31 Å to 5.05 Å without influencing the structure of the water channel of the H-pathway (Shimada, et al., 2017b). At 100 ms approximately 75% of CO has been released from Cu_B_^1+^. The residual CO at Cu_B_^1+^ at 100 μs is not given in the figure for the sake of simplicity. The CO release from Cu_B_^1+^ induced the closed to open transition in the water channel, giving 45% open and 55% closed structures. The coexistence of the open and closed structures in the X-ray structural results obtained at 100 μs was found by examination of the possibility of multiple structures. Ignoring the existence of the multiple structures would provide an unreal structure as in the case reported (Ishigam et al., 2017). At 100 μs, although 75% of the bound CO was released from Cu_B_^1+^, only 45% of the channels are in the open state, indicating that the response of the water channel against the O_2_ reduction site is not instantaneous, perhaps due to the slow structural changes in the 380-385 segment of the helix X. It is noteworthy that these dynamic aspects of the transition are undetectable without this time-resolved technique.

These results suggest critical roles of the Cu_B_ site in preventing back-leak of the pumping protons. In analogy to the CO binding to Cu_B_^1+^, CO binding to Fe_*a*3_^2+^ is unlikely to close the water channel instantaneously so that heme *a* oxidation, which triggers the proton pump, occurs before the water channel closure, giving back-leaks of pumping protons. However, the stoichiometric CO migration at 20 nsec as mentioned above indicates the absence of spontaneous reverse CO migration from Cu_B_^1+^ to Fe_*a*3_^2+^. In other words, in the CO binding process, direct CO binding to Fe_*a*3_^2+^ without forming Cu_B_^1+^-CO is highly unlikely. Furthermore, the stoichiometric CO migration from Fe_*a*3_^2+^ to Cu_B_^1+^ without the structural changes in the water channel suggests that the CO migration from Cu_B_^1+^ to Fe_*a*3_^2+^ occurs only when the water channel is closed. These structural findings suggest a mechanism of the water channel closure upon O_2_ binding as summarized in Figure 12B. One of the water molecules in the Mg-H_2_O cluster (water 10 in Figure 7) is hydrogen-bonded to one of the three histidine imidazole groups coordinated to Cu_B_ (Figure 12Ba). When the cluster is fully protonated (or has received four proton equivalents) (Figure 12Bb), the hydronium ion triggers a coordination state change in Cu_B_ to stimulate the O_2_ binding. Thus, O_2_ at Cu_B_ (Figure 12Bc) induces migration of the heme *a*_3_ plane to close the water channel through a structural relay system including the heme *a*_3_ vinyl group, Leu381, and Ser382. After the channel closure, the O_2_ is migrated to Fe_*a*3_^2+^ (Figure 12Bd). Then, the electron transfer from Fe_*a*_^2+^ is induced by the O_2_ bound at Fe_*a*3_^2+^, triggering the proton pump.

### 3.5 A simulation analysis for the Pm→Pr→F process for evaluation of the D-pathway mechanism

In the D-pathway mechanism, it is absolutely necessary to prevent delivery of the pumping protons to the O_2_-reduction site for avoiding dissipation of the proton-pumping energy. Thus, various mechanisms, designated as the “kinetic gating”, have been proposed, in which proton-pump is faster than the chemical proton transfer for the water formation. A kinetic gating mechanism has been extensively examined by multiscale simulations for the Pm→Pr→F process, one of the four proton-pumping steps in the catalytic cycle of CcO (Liang, et al., 2016; Liang, et al., 2017). A hydrophobic cavity (HC) is located above Glu242 connecting the O_2_-reduction site and the pumping-proton loading site, which was assigned to the propionate of the A-pyrrole ring of heme *a*_3_ by this simulation. The HC is dehydrated when Glu242 is protonated (or in the non-charged state) in the Pm form. Electron transfer from heme *a* to the Pm to produce the Pr drives the proton transfer from Glu242 to PLS. The resultant deprotonated E242 hydrates the HC to accelerate the proton transfer from E242 to the PLS. Rapid re-protonation of the E242, which dehydrate the HC, avoids reverse proton transfer from the PLS to the deprotonated E242. There is a large energy barrier in the Asn-gate region of the D-pathway including N98 and N80 against the reverse proton transfer to the N-side phase via the D-pathway (from E242 to D91). The calculated energy barrier of the decoupled mutants, N98T and N98S, are significantly lower than that of the wild type, giving the reverse proton transfer of the mutants from E244 to D91, in the Pr→F transition, much faster than that of the wild type. The calculated rates are even significantly faster than the proton pump process rate of the wild type enzyme (from the PLS to the P-side phase). Thus, the simulation results have been interpreted as follows: the reverse proton transfer from E242 to the N-side in the mutants are faster than the proton pump process of these mutant enzymes, assuming that the function of the proton pump system in these mutants is not influenced by these mutations, since the mutation sites are located far from the PLS. While the slow and essentially irreversible chemical proton transfer from E242 to the BNC would not be influenced by the increase in the reverse proton transfer, providing the decoupling (Liang, et al., 2017). However, the simulation report does not provide the forward proton transfer rate from D91 to E242 in the Pr→F transition. The energy barrier decrease in the Asn gate is highly likely to accelerate the forward proton transfer rate from D91 to E242 also, as in the case of the F→O transition, giving a normal transition (Liang, et al., 2017). The calculated forward rate is critical for evaluation of the simulation work.

As mentioned in Section 2.2, the decoupling mutation (N98D) clearly influences the X-ray structure of the E242 residue (Durr et al., 2008). The heme *a*_3_, which includes the PLS, the A-ring propionate, is located, not very far from E242. Thus, this X-ray structural result does not support this assumption for the integrity of the pumping system including the PLS in the decoupled mutant. These mutations could directly perturb the heme *a*_3_ structure to abolish the proton pumping function of the PLS. Even if the pumping proton transfer is faster than the chemical proton transfer in the D-pathway mechanism, the former must await (at the proton loading site) the arrival of the chemical proton to the O_2_-reduction site located near the proton loading site in order for pumping. Furthermore, since the direct transfer of the pumping proton from the proton loading site to the O_2_-reduction site would be highly exergonic, the blockage system must be strong. However, no positive experimental result suggesting this system has been reported thus far in our view. Kinetic gating is unnecessary if the proton transfer from PLS to BNC is structurally blocked. The simplest way for the blockage would be transportation of the pump protons through a pathway different form that for chemical protons as in the case of the H-pathway mechanism. Experimental trials for identification of hydration state changes in the HC are desirable.

## 4 Discussion, future experimental works

We reviewed the experimental accomplishments which have contributed significant improvements in our understanding of the reaction mechanism of CcO in the last 7 years or so. The important findings during these years may include as follows: 1) the X-ray structural demonstration that the Mg-H_2_O site has enough capacity for keeping four pumping protons and the water channel of the H-pathway is in the closed state in all the intermediate forms except for the R form, strongly supporting the H-pathway mechanism, 2) the X-ray crystallographic and VTVH-MCD spectroscopic findings for the intermediate forms supporting essentially irreversible nature of the proton pumping process, which provide high energy coupling efficiency in CcO. 3) the extensive simulation analyses for the Pm→Pr→F process of several decoupled mutant and wild type CcO for examination of the possible kinetic gating by energy barrier for preventing reverse proton transfer to the N-side. As described above, both the D- and H-pathway mechanisms coexist in this field, since no conclusive experimental evidence proving any of them has been obtained. In our view, the most important experimental evidence for the D-pathway mechanism is the structural finding proving blockage of the proton transfer from the PLS to the BNC, while that for the H-pathway mechanism is experimental identification of the pool site (or sites) for the pumping protons. Protons are pumped during these four transitions coupled with the proton-pump. In other words, the structures of the intermediate forms, P_m_, F, O, and E, themselves, do not have direct information on the proton pump process. The time-resolved analysis is absolutely desirable. The experimental conditions for the time-resolved X-ray structural analysis for bovine CcO has been established by the Rousseau group already (Ishigami et al., 2019). However, improvement of the crystallization conditions is necessary for providing resolution of the X-ray structural results sufficiently high for improvement of our understanding of the reaction mechanism. Another future works for the reaction mechanism studies would be 3D structural analyses for bacterial and yeast CcOs under various oxidation and ligand-binding states at high resolution and extensive mutational analyses of mammalian CcO including these decoupled mutations.

## Abbreviations

CcO, cytochrome *c* oxidase; heme *a* and heme *a*_3_, low and high-spin heme A molecules of CcO, respectively; Fe_*a*_ and Fe_*a*3_, iron ions of heme *a* and heme *a*_3_, respectively; Cu_A_ and Cu_B_, low and high potential copper sites of CcO, respectively; PDB, Protein Data Bank; BNC, O_2_-reduction site; PLS, proton loading site.

## References

[B1] Belevich I. Bloch D. A. Belevich N. Wikström M. Verkhovsky M. I. (2007). Exploring the proton pump mechanism of cytochrome *c* oxidase in real time. Proc. Natl. Acad. Sci. U. S. A*.* 104, 2685–2690. 10.1073/pnas.0608794104 17293458 PMC1796784

[B2] Belevich I. Gorbikova E. Belevich N. P. Rauhamäki V. Wikström M. Verkhovsky M. I. (2010). Initiation of the proton pump of cytochrome c oxidase. Proc. Natl. Acad. Sci. U. S. A. 107, 18469–18474. 10.1073/pnas.1010974107 20937896 PMC2972982

[B3] Belevich I. Verkhovsky M. I. Wikström M. (2006). Proton-coupled electron transfer drives the proton pump of cytochrome *c* oxidase. Nature 440, 829–832. 10.1038/nature04619 16598262

[B4] Bjorck M. L. Brzezinski P. (2018). Control of transmembrane charge transfer in cytochrome *c* oxidase by the membrane potential. Nat. Com. 9, 3187. 10.1038/s41467-018-05615-5 PMC6085328 30093670

[B5] Bjorck M. L. Vihjalmsdottir J. Hartley A. M. Meunier B. Ojemyr L. N. Marechal A. (2019). Proton-transfer pathways in the mitochondrial *S. cerevisiae* cytochrome c oxidase. Sci. Rep. 9, 20207. 10.1038/s41598-019-56648-9 31882860 PMC6934443

[B6] Bloch D. Belevich I. Jasaitis A. Ribacka C. Puustinen A. Verkhovsky M. I. (2004). The catalytic cycle of cytochrome c oxidase is not the sum of its two halves. Proc. Natl. Acad. Sci. U. S. A*.* 101, 529–533. 10.1073/pnas.0306036101 14699047 PMC327181

[B7] Brändén G. Gennis R. B. Peter Brzezinski P. (2006). Transmembrane proton translocation by cytochrome c oxidase. Biochim. Biophys. Acta 1757, 1052–1063. 10.1016/j.bbabio.2006.05.020 16824482

[B8] Cai X. Haider K. Lu J. Radic S. Son C. Y. Cui Q. (2018). Network analysis of a proposed exit pathway for protons to the P-side of cytochrome c oxidase. Biochim. Biophys. Acta 1859, 997–1005. 10.1016/j.bbabio.2018.05.010 29778689

[B9] Capitanio N. Capitanio G. Boffoli D. Sergio Papa S. (2000a). The proton/electron coupling ratio at heme *a* and Cu_A_ in bovine heart cytochrome *c* oxidase. Biochemistry 39, 15454–15461. 10.1021/bi001940z 11112531

[B10] Capitanio N. Minuto M. De Nitto E. Palese L. L. Nicholls P. (2000b). Coupling of electron transfer with proton transfer at heme a and CuA (redox bohr effects) in cytochrome cOxidase. Studies with the carbon monoxide inhibited enzyme. Biochemistry 39, 6373–6379. 10.1021/bi0003137 10828951

[B11] Caughey W. S. Wallace W. J. Volpe J. A. Yoshikawa S. (1976). Cytochrome *c* Oxidase” in *The Enzymes* 3^rd^ e. Editor Boyer P. D. (New York: Academic Press), Vol. 13, 299–344.

[B12] Dodson E. D. Zhao X. J. Caughey W. S. Elliott C. M. (1996). Redox dependent interactions of the metal sites in carbon monoxide-bound cytochrome c oxidase monitored by infrared and UV/visible spectroelectrochemical methods. Biochemistry 35, 444–452. 10.1021/bi951313n 8555214

[B13] Durr K. L. Koepke J. Hellwig P. Muller H. Angerer H. Peng G. (2008). A D-pathway mutation decouples the *Parcoccus denitrifican* cytochrome *c* oxidase by altering the side-chain orientation of a distant conserved glutamate. J. Mol. Biol. 384, 865–877. 10.1016/j.jmb.2008.09.074 18930738

[B14] Faxén K. Gilderson G. Adelroth P. Brzezinski P. (2005). A mechanistic principle for proton pumping by cytochromr *c* oxidase. Nature 437, 286–289. 10.1038/nature03921 16148937

[B15] Forte E. Barone M. C. Brunori M. Sarti P. Giuffrè A. (2002). Redox-linked protonation of cytochrome *c* oxidase: The effect of chloride bound to Cu_B_ . Biochemistry 41, 13046–13052. 10.1021/bi025917k 12390032

[B16] Han S. Takahashi S. Rousseau D. (2000). Time dependence of the catalytic intermediates in cytochromec oxidase. J. Biol. Chem. 275, 1910–1919. 10.1074/jbc.275.3.1910 10636892

[B17] Isaacs N. S. (1995). Acids and bases, electrophiles and nucleophiles” in physical organic Chemistry. 2nd ed Longman Scientific and Technical: U. K), 235–286.

[B18] Ishigam I. Zatsepin N. A. Hikita M. Conrad C. E. Nelson G. Coe J. D. (2017). Crystal structure of CO-bound cytochrome*c* oxidase determined by serial femtosecond X-ray crystallography at room temperature. Proc. Natl. Acad. Sci. U. S. A. 114, 8011–8016. 10.1073/pnas.1705628114 28698372 PMC5544322

[B19] Ishigami I. Lewis-Ballester A. Echelmeier A. Brehm G. Zatsepin N. A. Grant T. D. (2019). Snapshot of an oxygen intermediate in the catalytic reaction of cytochrome c oxidase. Proc. Natl. Acad. Sci. U. S. A. 116, 3572–3577. 10.1073/pnas.1814526116 30808749 PMC6397517

[B20] Jancura D. Antalik M. Vladimir Berka V. Palmer G. Fabian M. (2006). Filling the catalytic site of cytochrome *c* oxidase with electrons. Reduced Cu_B_ facilitates internal electron transfer to heme *a* _3_ . J. Biol. Chem. 281, 20003–20010. 10.1074/jbc.m602066200 16704969

[B21] Jasaitis A. Backgren C. Morgan J. E. Puustinen A. Verkhovsky M. I. Wikström M. (2001). Electron and proton transfer in the arginine-54-methionine mutant of cytochrome *c* oxidase from *Paracoccus denitrificans* . Paracoccus denitrificans Biochem. 40, 5269–5274. 10.1021/bi002948b 11318650

[B22] Jose A. Schaefer A. W. Roveda A. C. Jr Transue W. J. Choi S. K. Ding Z. (2021). The three-spin intermediate at the O–O cleavage and proton-pumping junction in heme–Cu oxidases. Science 373, 1225–1229. 10.1126/science.abh3209 34516790 PMC9036946

[B23] Jünemann S. Heathcote P. Rich P. R. (2000). The reactions of hydrogen peroxide with bovine cytochrome *c* oxidase. Biochim. Biophys. Acta - Bioenerg. 1456, 56–66. 10.1016/s0005-2728(99)00105-x 10611456

[B24] Kim H. Rogler P. J. Savita P. J. SharmaAndrewSchaefer K. W. A. W. Solomon E. I. Karlin K. D. (2020). Heme-Fe^III^ superoxide, seroxide and hydroperoxide thermodynamic relationships: Fe^III^-O_2_ ^•−^ complex H-atom abstraction reactivity. J. Am. Chem. Soc. 142, 3104–3116. 10.1021/jacs.9b12571 31913628 PMC7034651

[B25] Kolbe F. Safarian S. Piórek Z. Welsch H. Müller H. Michel H. (2021). Cryo-EM structures of intermediates suggest an alternative catalytic reaction cycle for cytochrome c oxidase. Nat. Comm. 12, 6903–6913. 10.1038/s41467-021-27174-y PMC8617209 34824221

[B26] Lee H. M. Das T. K. Rousseau D. L. Mills D. Ferguson-Miller S. Gennis R. B. (2000). Mutations in the putative H-channel in the cytochrome c oxidase from Rhodobacter sphaeroides show that this channel is not important for proton conduction but reveal modulation of th properties of heme *a* . Biochemistry 39, 2989–2996. 10.1021/bi9924821 10715119

[B27] Lepp H. Svahn E. Faxén K. Brzezinski P. (2008). Charge transfer in the K proton pathway linked to electron transfer to the catalytic site in cytochrome c oxidase. Biochemistry 47, 4929–4935. 10.1021/bi7024707 18393448

[B28] Liang R. Swanson J. M. J. Peng X. Wikstrom M. Voth G. A. (2016). Multiscale simulations reveal key features of the proton-pumping mechanism in cytochrome *c* oxidase. Proc. Natl. Acad. Sci. U. S. A. 113, 7420–7425. 10.1073/pnas.1601982113 27339133 PMC4941487

[B29] Liang R. Swanson J. M. J. Wikstrom M. Voth G. A. (2017). Understanding the essential proton-pumping kinetic gates and decoupling mutations in cytochrome *c* oxidase. Proc. Natl. Acad. Sci. U. S. A. 114, 5924–5929. 10.1073/pnas.1703654114 28536198 PMC5468613

[B30] Maréchal A. Xu J-Y. Hartley A. M. Haraux F. Meunier B. (2020). A common coupling mechanism for A-type heme-copper oxidases from bacteria to mitochondria. Proc. Natl. Acad. Sci. U. S. A. 117, 9349–9355. 10.1073/pnas.2001572117 32291342 PMC7196763

[B31] Mochizuki M. Aoyama H. Shinzawa-Itoh K. Usui T. Tsukihara T. Yoshikawa S. (1999). Quantitative reevaluation of the redox active sites of crystalline bovine heart cytochrome c oxidase. J. Biol. Chem*.* 274, 33403–33411. 10.1074/jbc.274.47.33403 10559221

[B32] Ogura T. Hirota S. Proshlyakov D. A. Shinzawa-itoh K. Yoshikawa S. Kitagawa T. (1996). Time resolved resonance Raman evidence for tight coupling between electron transfer and proton pumping of cytochrome *c* oxidase upon the change from the Fe^V^ oxidation level to the Fe^IV^ oxidation level. J. Am. Chem. Soc*.* 118, 5443–5449. 10.1021/ja951922i

[B33] Ogura T. Takahashi S. Hirota S. Shinzawa-Itoh K. Yoshikawa S. Appelman E. H. (1993). Time-resolved resonance Raman elucidation of the pathway for dioxygen reduction by cytochrome c oxidase. J. Am. Chem. Soc. 115, 8527–8536. 10.1021/ja00072a002

[B34] Pawate A. S. Morgan J. Namslauer A. Mills D. Brzezinski P. Ferguson-Miller S. (2002). A mutation in subunit I of cytochrome oxidase from *Rhodobacter sphaeroides* results in an increase in steady-state activity but completely eliminates proton pumping. Biochemistry 41, 13417–13423. 10.1021/bi026582+ 12416987

[B35] Perrin C. L. (1989). Proton exchange in amides: Surprises from simple systems. Acc. Chem. Res. 22, 268–275. 10.1021/ar00164a002

[B37] Poiana F. von Ballmoos C. Gonska N. Blomberg M. R. A. Ädelroth P. Brzezinski P. (2017). Splitting of the O–O bond at the heme-copper catalytic site of respiratory oxidases. Sci. Adv. 3, e1700279. 10.1126/sciadv.1700279 28630929 PMC5473675

[B38] Popovic D, M. Stuchebrukhov A. A. (2012). Coupled electron and proton transfer reactions during the O→E transition in bovine cytochrome c oxidase. Biochimica Biophysica Acta 1817, 506–517. 10.1016/j.bbabio.2011.10.013 PMC4220735 22086149

[B39] Popovic D, M. Stuchebrukhov A. A. (2005). Proton exit channels in bovine cytochrome *c* oxidase. J. Phys. Chem. B 109, 1999–2006. 10.1021/jp0464371 16851184

[B40] Popovic D. M. (2013). Current advances in research of cytochrome *c* oxidase. Amino Acids 45, 1073–1087. 10.1007/s00726-013-1585-y 23999646

[B41] Popovic D. M. Leontyev I. V. Beech D. G. Stuchebrukhov A. A. (2010). Similarity of cytochrome c oxidases in different organisms. Proteins 78, 2691–2698. 10.1002/prot.22783 20589635 PMC4220736

[B42] Sharma V. Jambrina P. G. Kaukonen M. Rosta E. Rich P. R. (2017). Insights into functions of the H-channel of cytochrome *c* oxidase from atomistic molecular dynamic simulations. Proc. Natl. Acad. Sci. U. S. A. 114, E10339–E10348. 10.1073/pnas.1708628114 29133387 PMC5715751

[B43] Shimada A. Hatano K. Tadehara H. Yano N. Shinzawa-Itoh K. Yamashita E. (2018). X-ray structural analyses of azide-bound cytochrome *c* oxidases reveal that the H-pathway is critically important for the proton-pumping activity. J. Biol. Chem. 293, 14868–14879. 10.1074/jbc.ra118.003123 30077971 PMC6153300

[B44] Shimada A. Etoh Y. Kitoh-Fujisawa R. Sasaki A. Shinzawa-Itoh K. Hiromoto T. (2020). X-ray structures of catalytic intermediates of cytochrome c oxidase provide insights into its O_2_ activation and unidirectional proton-pump mechanismsc oxidase provide insights into its O_2_ activation and unidirectional proton-pump mechanisms. J. Biol. Chem. 295. 5818–5833. 10.1074/jbc.ra119.009596 PMC7186171 32165497

[B45] Shimada A. Hara F. Shinzawa-Itoh K. Nobuko Kanehisa N. Yamashita E. Muramoto K. (2021). Critical roles of the Cu_B_ site in efficient proton pumping as revealed by crystal structures of mammalian cytochrome *c* oxidase catalytic intermediates. J. Biol. Chem*.* 297, 100967–100982. 10.1016/j.jbc.2021.100967 34274318 PMC8390519

[B46] Shimada A. Kubo M. Baba S. Yamashita K. Hirata K. Ueno G. (2017a). A nanosecond time-resolved XFEL analysis of structural changes associated with CO release from cytochrome c oxidase. Sci. Adv. 3, e1603042. 10.1126/sciadv.1603042 28740863 PMC5510965

[B47] Shimada S. Shinzawa-Itoh K. Baba J. Aoe S. Shimada A. Yamashita E. (2017b). Complex structure of cytochrome c-cytochrome c oxidase reveals a novel protein-protein interaction mode. EMBO J. 36, 291–300. 10.15252/embj.201695021 27979921 PMC5286356

[B48] Shimokata K. Katayama Y. Murayama H. Suematsu M. Tsukihara T. Muramoto K. (2007). The proton pumping pathway of bovine heart cytochrome *c* oxidase. Proc. Natl. Acad. Sci. U. S. A. 104, 4200–4205. 10.1073/pnas.0611627104 17360500 PMC1820732

[B49] Shinzawa-Itoh K. Hatanaka K. Fujita K. Yano N. Ogasawara Y. Iwata J. (2021). The 1.3-Å resolution structure of bovine cytochrome c oxidase suggests a dimerization mechanism. BBA Adv. 1, 100009. 10.1016/j.bbadva.2021.100009 37082008 PMC10074962

[B50] Tsukihara T. Shimokata K. Katayama Y. Shimada H. Muramoto K. Aoyama H. (2003). The low-spin heme of cytochrome *c* oxidase as the driving element of the proton-puming process. Proc. Natl. Acad. Sci. U. S. A. 100, 15304–15309. 10.1073/pnas.2635097100 14673090 PMC307562

[B51] Verkhovsky M. I. Jasaitis A. Verkhovskaya M. L. Morgan J. E. Wikström M. (1999). Proton translocation by cytochrome *c* oxidase. Nature 400, 480–483. 10.1038/22813 10440381

[B52] Wikström M. Krab K. Sharma V. (2018). Oxygen activation and energy conservation by cytochrome c oxidase. Chem. Rev. 118, 2469–2490. 10.1021/acs.chemrev.7b00664 29350917 PMC6203177

[B53] Wilson D. F. Lindsay J. G. Brocklehurst E. S. (1972). Heme-heme interaction in cytochrome oxidase. Biochim. Biophys. Acta - Bioenerg*.* 256, 277–286. 10.1016/0005-2728(72)90058-8 4335838

[B54] Yano N. Muramoto K. Shimada A. Takemura S. Baba J. Fujisawa H. (2016). The Mg^2+^-containing water cluster of mammalian cytochrome c oxidase collects four pumping proton equivalents in each catalytic cycle. J. Biol. Chem. 291, 23882–23894. 10.1074/jbc.m115.711770 27605664 PMC5104913

[B55] Yoshikawa S. Shimada A. (2015). Reaction mechanism of cytochrome c oxidase. Chem. Rev. 115, 1936–1989. 10.1021/cr500266a 25603498

[B56] Zaslavsky D. Kaulen A. D. Smirnova I. A. Vygodina T. Konstantinov A. A. Belozersky A. N. (1993). Flash-induced membrane potential generation by cytochrome c oxidase. FEBS Lett. 336, 389–393. 10.1016/0014-5793(93)80843-j 8282099

[B57] Zaslavsky D. Sadoski R. C. Rajagukguk S. Geren L. Millett F. Durham B. (2004). Direct measurement of proton release by cytochrome *c* oxidase in solution during the F→ O transition. Proc. Natl. Acad. Sci. U. S. A*.* 101, 10544–10547. 10.1073/pnas.0401521101 15247424 PMC489974

[B58] Zaslavsky D. Smirnova I. A. Ädelroth P. Brzezinski P. (1999). Observation of a novel transient ferryl complex with reduced CuB in cytochrome c oxidase. Biochemistru 38, 2307–2311. 10.1021/bi9822832 10029523

